# Effects of prenatal stress on neuroactive steroid responses to acute stress in adult male and female rats

**DOI:** 10.1111/jne.12916

**Published:** 2020-12-03

**Authors:** Ying Sze, Paula J. Brunton

**Affiliations:** ^1^ Centre for Discovery Brain Sciences University of Edinburgh Edinburgh UK; ^2^ The Roslin Institute University of Edinburgh Edinburgh UK; ^3^ Zhejiang University‐University of Edinburgh Joint Institute Haining China

**Keywords:** allopregnanolone, early‐life stress, hypothalamic‐pituitary‐adrenal axis, sex differences, tetrahydrodeoxycorticosterone

## Abstract

Acute swim stress results in the robust production of several neuroactive steroids, which act as mediators of the stress response. These steroids include glucocorticoids, and positive GABA_A_ receptor modulatory steroids such as allopregnanolone and tetrahydrocorticosterone (THDOC), which potentiate inhibitory GABA signalling, thereby playing a role in the negative control of the hypothalamic‐pituitary‐adrenal (HPA) axis. Prenatally stressed (PNS) offspring exhibit increased vulnerability to stress‐related disorders and frequently display exaggerated HPA axis responses to stressors during adulthood, which may be a result of reduced neuroactive steroid production and consequently inhibitory signalling. Here, we investigated whether exposure of rats to prenatal social stress from gestational day 16‐20 altered neuroactive steroid production under non‐stress conditions and in response to an acute stressor (swim stress) in adulthood. Using liquid chromatography‐mass spectrometry, nine neuroactive steroids were quantified (corticosterone, deoxycorticosterone [DOC], dihydrodeoxycorticosterone, THDOC, progesterone, dihydroprogesterone, allopregnanolone, pregnenolone, testosterone) in plasma and in five brain regions (frontal cortex, hypothalamus, amygdala, hippocampus, brainstem) of male and female control and PNS rats. There was no difference in the neuroactive steroid profile between control and PNS rats under basal conditions. The increase in circulating corticosterone induced by acute swim stress was similar in control and PNS offspring. However, greater stress‐induced corticosterone and DOC concentrations were observed in the brainstem of male PNS offspring, whereas DOC concentrations were lower in the hippocampus of PNS females compared to controls, following acute stress. Although PNS rats did not show deficits in allopregnanolone responses to acute stress, there were modest deficits in the production of THDOC in the brainstem, amygdala, and frontal cortex of PNS males and in the frontal cortex of PNS females. The data suggest that neuroactive steroid modulation of GABAergic signalling following stress exposure may be affected in a sex‐ and region‐specific manner in PNS offspring.

## Graphical abstract

Central and peripheral neuroactive steroid concentrations were quantified under basal conditions and in response to acute stress in the adult male and female offspring of rats born to mothers that either experienced social stress or remained undisturbed during their pregnancy. We report sex‐ and region‐dependent deficits in the central production of the GABA_A_ receptor modulatory steroids, dihydrodeoxycorticosterone and tetrahydrocorticosterone in response to acute stress in prenatally stressed offspring, which may contribute to the altered stress responsivity and increased vulnerability to stress‐related behaviours, previously demonstrated in these animals.

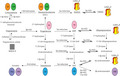

## 1 INTRODUCTION

**Figure 1:**
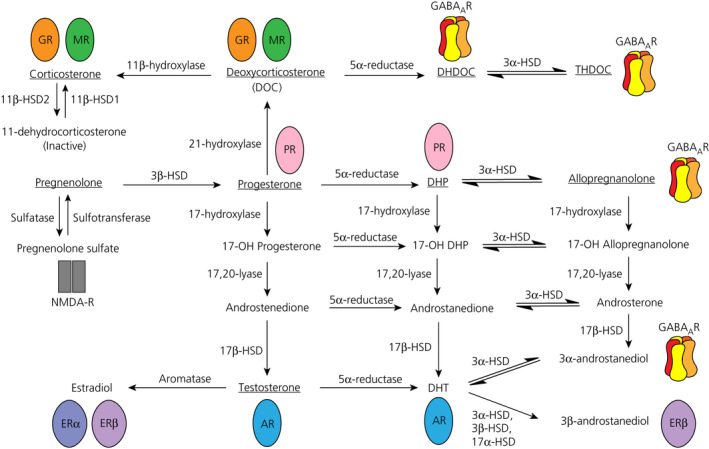
Steroid interconversion and their receptors. The steroids investigated in the present study are underlined, shown alongside the receptors through which they exert their rapid effects. All neuroactive steroids are produced from pregnenolone, which can be converted to progesterone by 3β‐hydroxysteroid hydrogenase (3β‐HSD), or sulphated to pregnenolone sulphate, which positively modulates NMDA receptor (NMDA‐R) signalling. Progesterone can subsequently be converted to deoxycorticosterone (DOC) then corticosterone, and both target glucocorticoid and mineralocorticoid receptors (GR and MR, respectively). Progesterone and DOC can also undergo 5α‐reduction to respectively form dihydroprogesterone (DHP), which has a greater affinity for the progesterone receptor (PR), or dihydrodeoxycorticosterone (DHDOC), which modulates GABA_A_ receptors (GABA_A_R). Following a further 3α‐hydroxysteroid dehydrogenase (3α‐HSD) reduction, the 5α,3α‐reduced steroids, allopregnanolone and tetrahydrodeoxycorticosterone (THDOC) are formed, both of which are positive modulators of the GABA_A_R. Progesterone can be converted to androgens by a two‐step pathway, catalysed by the 17‐hydroxylase and 17,20‐lyase activities of a single enzyme, cytochrome P450c17. Dihydrotestosterone (DHT), which has more potent effects on the androgen receptor (AR) compared to testosterone, is produced from testosterone via 5α‐reduction or from a ‘backdoor pathway’ without the need for testosterone production. DHT is subsequently reduced to form 3α‐androstanediol (3α‐diol) and 3β‐androstanediol (3β‐diol). Although 3α‐diol potentiates GABA_A_ receptors, 3β‐diol is likely to exert its rapid effects through the oestrogen receptor β (ERβ). Lastly, testosterone can be converted to 17β‐oestradiol via the actions of aromatase

Several steroids are rapidly produced as a physiological response to acute stress, where they act as allostatic mediators to allow the body to adapt and cope with the stressor.^1^ As well as the production of glucocorticoids (ie, cortisol in humans, corticosterone in rats and mice) following activation of the hypothalamic‐pituitary‐adrenal (HPA) axis, other steroids are also produced, such as deoxycorticosterone (DOC), progesterone and their metabolites, with elevated levels detected in both the periphery and in the brain following acute stress.^2^, ^3^, ^4^ Steroids that exert rapid non‐genomic effects in the brain are collectively known as neuroactive steroids,^5^ and they can originate from peripheral endocrine organs or are produced de novo via the action of steroidogenic enzymes such as 5α‐reductase and 3α‐hydroxysteroid dehydrogenase (3α‐HSD) expressed within the brain.^6^, ^7^ Neuroactive steroids exert rapid effects by binding to their membrane‐bound cognate receptors^8^ or to ion channel‐associated membrane receptors such as GABA^9^ or glutamate NMDA receptors,^10^ and contribute to the rapid modulation of the stress response by fine‐tuning neuronal signalling^11^ (Figure 1). For example, although glucocorticoids are known for their peripheral actions on glucose metabolism, they also bind to central glucocorticoid (GR) and mineralocorticoid (MR) receptors to modulate synaptic transmission, resulting in negative‐feedback regulation of the HPA axis.^12^, ^13^ Neuroactive steroids that positively modulate GABA_A_ receptors, such as the progesterone metabolite, allopregnanolone, and the corticosterone metabolite, tetrahydrodeoxycorticosterone (THDOC), are of particular interest because their increase in the brain following acute stress has been proposed to be necessary for both mounting the stress response,^14^ as well as for terminating the HPA axis response when it is no longer needed, by increasing inhibitory signalling.^15^, ^16^

Evidence from human and animal studies demonstrate that exposure to early‐life stress can alter the ability of an individual to cope with challenging events later in life.^17^, ^18^, ^19^ The vulnerability to stress‐related affective disorders, such as anxiety and depression, is also increased and is frequently accompanied by HPA axis dysregulation.^20^, ^21^ In our model of prenatal social stress, the male prenatally stressed (PNS) offspring exhibit an anxious phenotype, and both sexes display greater HPA axis activation in response to acute stressors such as systemic interleukin (IL)‐1β administration and restraint.^22^, ^23^ These phenotypes can be considered as a manifestation of “allostatic overload”, where physiological systems fail to adapt to perturbations, resulting in vulnerability instead of resilience when faced with stressors.^1^ Allostatic overload arises from an imbalance in the production or action of allostatic mediators.^1^ Therefore, it is possible that the steroidal milieu of PNS offspring differs from that of non‐stressed offspring, particularly so for positive GABA_A_ modulatory neuroactive steroids, given their role in ameliorating anxious behaviour and HPA axis dysregulation associated with prenatal stress.^22^, ^24^, ^25^ The reduced production of these steroids, would be expected to lead to a general loss of inhibitory tone, and in turn greater and/or more prolonged HPA axis activation that may increase vulnerability to stress‐related disorders.^16^, ^26^

Indeed, there is evidence that supports the concept of deficits in neuroactive steroid production in PNS offspring.^22^, ^27^ The capacity for neurosteroid production is evidently reduced, as reflected by lower levels of 5α‐reductase gene expression and activity in the brain.^22^, ^28^ Moreover, up‐regulating 5α‐reductase and 3α‐HSD gene expression in the brainstem (using adenoviruses), or administration of allopregnanolone to female PNS offspring or 3β‐androstanediol to male PNS offspring, respectively, normalises dysregulated HPA axis responses to systemic IL‐1β administration.^22^ Together, these data suggest that deficient endogenous production of neuroactive steroids may underlie the exaggerated HPA axis stress responses in PNS offspring; however, neuroactive steroid concentrations have never been directly quantified in PNS offspring in response to stress in later life. Therefore, the present study aimed to compare the neuroactive steroid milieu of control and PNS offspring under basal conditions, as well as following exposure to an acute stressor. Specifically, we quantified nine neuroactive steroids (Figure 1) that are known mediators of the stress response, including corticosterone, the end product of HPA axis activation; the positive GABA_A_ receptor modulators, allopregnanolone and THDOC; their precursors, dihydroprogesterone (DHP) and progesterone, as well as dihydrodeoxycorticosterone (DHDOC) and DOC, respectively; and lastly testosterone, which contributes to sex differences in the stress response.^29^ These steroids were measured in five distinct brain regions: the hypothalamus, where stress‐related circuitry are integrated; the hippocampus, amygdala and the prefrontal cortex, which are limbic areas that together process stressful experiences; and the brainstem, an area that receives inputs regarding homeostatic perturbations.^30^ Given allopregnanolone and THDOC potentiate the inhibitory effects of GABA on corticotropin‐releasing hormone (CRH) neurones to decrease activity of the HPA axis,^14^, ^16^, ^31^ we hypothesised that the concentrations of these 3α,5α‐reduced GABA_A_ modulatory neuroactive steroids would be lower in the brains of PNS offspring compared to control offspring following acute stress.

## 2 MATERIALS AND METHODS

### 2.1 Animals

Female Sprague‐Dawley rats used for generating control and PNS offspring were purchased from Charles River (Margate, UK). All rats were group‐housed in individually ventilated cages (IVCs), under a 12:12 hour light/dark photocycle (lights on at 08.00 am) at 22 ± 1˚C and 58 ± 3% relative humidity, and with access to drinking water and standard rodent diet (Harlan Teklad) available ad lib., unless otherwise stated. Breeding females were fed a 50:50 mixture of 14% and 19% protein diet (Harlan Teklad) throughout pregnancy and lactation. All animal experiments were approved by the local Animal Welfare and Ethical Review Body and performed in accordance with the UK Animals (Scientific Procedures) Act 1986 and the European Directive (2010/63/EU).

### 2.2 Generation of prenatally stressed rats using the modified resident‐intruder paradigm

Female rats (n = 20) were housed with a sexually experienced male, and mating was confirmed by the presence of a semen plug in the breeding cage the next morning. Female rats were returned to same‐sex group‐housing after mating (designated as gestational day (GD) 1) until GD16, after which time they were housed singly in IVCs. Lactating dams (ie, ‘residents’ for the resident‐intruder paradigm; n = 10) were also generated, by mating them 1 week ahead of the experimental dams. Lactating ‘resident’ rats were group‐housed until GD20, then transferred to individual open top cages in a separate room in the animal facility.

Half of the experimental pregnant dams (n = 10) were exposed to social stress from GD16 to GD20 using the modified resident‐intruder paradigm, as described previously.^23^ Experimental pregnant females (‘intruders’) were weighed and then transferred to the home cage of the lactating ‘residents’, for 10 minutes each day from GD16 to GD20, between 10.00 am and 2.00 pm. In each case, pregnant dams (‘intruders’) were paired with an unfamiliar lactating ‘resident’ to prevent habituation. Experimental pregnant dams were returned to their home cage immediately after the 10 minutes of social stress, whereas pregnant control dams (n = 10) remained in their home cages throughout gestation, except for daily weighing from GD16 to GD20.

Following birth, the control and PNS offspring remained with their mothers until weaning on PND23. At weaning, two male and two female rats from each litter were tail‐marked and housed in same‐sex groups with other rats of the same prenatal stress status from other litters.

### 2.3 Acute swim stress

Swim stress was selected as the acute stressor because it has previously been shown to robustly increase the concentrations of neuroactive steroids such as allopregnanolone and pregnenolone.^3^, ^4^, ^32^

**Figure 2:**
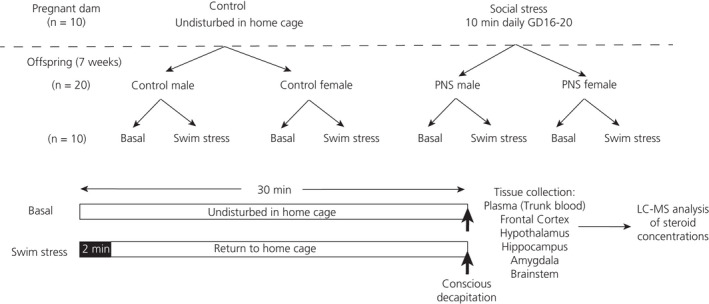
Experimental design. Control and prenatally stressed (PNS) offspring were generated from non‐stressed and socially stressed pregnant dams, respectively. At 7 weeks old, the male and female control and PNS rats were further divided into basal and acute swim stress groups. Rats were killed by conscious decapitation for tissue collection and liquid chromatography‐mass spectrometry (LC‐MS) analysis of steroid concentrations. GD, gestational day

At approximately 7 weeks of age (actual range = 49‐52 days old), male and female (randomly cycling) control and PNS offspring (each from a different litter; n = 10 per group) were exposed to an acute swim stress (Figure 2). Swim‐stressed rats were placed in a glass cylinder (diameter 25 cm, height 50 cm) filled with water (22‐23°C) to a depth of 30 cm. After 2 minutes of swimming, rats were gently towel dried, then returned to their home cage. Swim‐stressed rats were killed in a separate room 30 minutes after the onset of swim stress by conscious decapitation. Non‐swim‐stressed (ie basal) groups (n = 10 per group each from a different litter) remained undisturbed in their home cages before being rapidly transferred to the separate room for conscious decapitation (Figure 2). All procedures were carried out between 10.00 am and 2.00 pm because, during this time period, the evidence indicates oestrus cycle stage has very little effect on brain concentrations of pregnenolone, progesterone, DHP or allopregnanolone.^33^

### 2.4 Tissue collection

Trunk blood (approximately 5 mL) was collected into a chilled tube containing 0.5 mL of 5% (w/v) ethylenediaminetetracaetic acid and kept on ice until centrifugation at 4°C to separate the plasma. Plasma was stored at −20°C until the steroid assays were performed. The brain was rapidly removed and one hemisphere was subjected to gross dissection to remove the frontal cortex, hypothalamus, hippocampus, amygdala and brainstem, as described previously.^4^ Dissected brain tissue was frozen immediately on dry ice, and stored at −80°C until sample processing for liquid chromatography‐tandem mass spectroscopy (LC‐MS/MS).

### 2.5 LC‐MS/MS

LC‐MS/MS was carried out as described previously,^4^ with minor modifications, including the additional quantification of THDOC and the alteration of the standard calibration range from 102.4‐25 000 pg mL^‐1^ for all steroid analytes. Corticosterone, deoxycorticosterone, 5α‐dihydrodeoxycorticoserone, progesterone, 5α‐dihydroprogesterone, allopregnanolone, pregnanolone, testosterone (Steraloids Inc., Newport, RI, USA) and 5α,3α‐THDOC (Sigma, St Louis, MO, USA) were first dissolved in methanol, then combined into a 25 ng mL^‐1^ solution in 4% (w/v) bovine serum albumin (BSA) (VWR Scientific, Radnor, PA, USA). Standards of 25 ng mL^‐1^ were serially diluted 2.5‐fold in 4% BSA, generating seven calibration points: 10 ng mL^‐1^, 4 ng mL^‐1,^ 1.6 ng mL^‐1^, 640 pg mL^‐1^, 256 pg mL^‐1^ and 102.4 pg mL^‐1^. 100 μL of the standard calibrant solution was used for processing. The deuterated internal standards progesterone‐D9 (#Q2600‐014; Steraloids Inc.), allopregnanolone‐D5 (#5532; Tocris Bioscience, St Louis, MO, USA) and corticosterone‐D5 (#802905; Sigma) were combined and diluted into a working solution of 25 ng mL^‐1^ in 50% methanol and 20 μL of this deuterated internal standard mix was added to each sample.

Frozen brain samples were weighed prior to sample processing, and all samples from the same brain region were processed and analysed in the same day. The mean ± SD weights of tissues were: 29.9 ± 0.9 mg for frontal cortex; 46.4 ± 1.2 mg for hypothalamus; 21.6 ± 0.7 mg for amygdala; 53.6 ± 0.9 mg for hippocampus; and 99.5 ± 2.0 mg for brainstem. Each batch of samples was processed with seven standard calibrants and a zero sample containing only 4% BSA. For standard calibrants and plasma, 100 μL was used. Samples were homogenised in 500 µL of methanol/1% formic acid (FA) and sonicated on ice. For standard calibrants and plasma, 400 µL of methanol/1% FA was added. Next, 20 μL of deuterated internal standard mix (25 ng mL^‐1^ of each internal standard) was added and homogenates were briefly sonicated. After incubation on dry ice for 30 minutes to aid protein precipitation, homogenates were centrifuged for 10 min (13 000 *g* at 4°C). The supernatant was decanted into a borosilicate tube and the pellet was homogenised and sonicated again with another 500 µL of methanol/1% FA. After centrifugation, supernatants were combined from the two rounds of extraction, then diluted with LC‐MS grade water to a final methanol concentration of 30%.

Steroids in both plasma and brain samples were extracted by solid phase extraction using C18 columns (Supelco Discovery DSC‐18 SPE Cartridge; Sigma; #52602‐U, bed weight 100 mg) as described previously.^4^ Columns were activated with 1 mL of methanol and equilibrated with another 1 mL of 30% methanol. Diluted supernatants from homogenates (3 mL; with methanol concentration of 30%) were then loaded, followed by two 1‐mL washes of 50% methanol. All steps were assisted by centrifugation at 50 g (average flow rate of 0.5 mL min^‐1^). Steroids were eluted with 1 mL of 85% methanol by gravity flow. The collected eluate was dried in a vacuum overnight. The method of extraction was optimised (see Supporting information, Figure S1) and yielded a good recovery rate of 73%‐108%.^4^

On the day of LC‐MS analysis, 400 μL of freshly prepared derivatisation agent (1 mg mL^‐1^ of Girard's T reagent; Sigma, #89397; dissolved in methanol containing 0.2% FA) was added to the dried samples. After incubation at 37°C for 30 minutes, the reaction was stopped by the addition of 50 μL of 5% ammonium hydroxide (ACROS Organics, Faiir Lawn, NJ, USA) in methanol. Samples were dried in a vacuum dryer, then reconstituted in 50 μL of 50% methanol for analysis.

LC‐MS analysis was performed using an Ultimate 3000 Dionex high‐performance liquid chromatography (HPLC) system (Thermo Fisher, Waltham, MA, USA) with a refrigerated autosampler (8°C), coupled to an AmaZon ETD ion trap mass spectrometer (Bruker Daltonics, Billerica, MA, USA). Briefly, separation of steroids on reverse phase HPLC was achieved on the ACE UltraCore 2.5 µmol L^‐1^ Super C18 column (Advanced Chromatography Technologies, Aberdeen, UK), maintained at 40°C (75 mm by 2.1 mm inner diameter; #CORE‐25A‐7502U; Advance Chromatography Technologies). Mobile phase A consisted of 50 mmol L^‐1^ ammonium formate pH 3 and mobile phase B consisted of methanol with 0.1% FA. Steroids were analysed simultaneously using multiple reaction monitoring, with positive electrospray ionisation and collision‐induced fragmentation. Representative chromatograms (see Supporting information, Figures S2 and S3), gradient characteristics for LC (see Supporting information, Figure S4), and transitions monitored for MS (see Supporting information, Table S1) are provided. Data were acquired using hystar (Bruker Daltonics) and the peak area under curve (AUC) was extracted and automatically integrated using quantanalysis, version 2.0 (Bruker Daltonics). The ratio of the peak AUC of the target analyte and the peak AUC of the respective internal standards was used to construct the calibration curve, with linear regression and a weighting of 1/×. Calibration curves were all linear and a quality control check using low, medium and high concentrations of steroid analytes was carried out prior to the study, where the intra‐ and inter‐assay variability were within acceptable limits (see Supporting information, Table S2). Values obtained using this method were comparable to those reported by us previously^4^ and other studies utilising gas chromatography‐MS^32^, ^34^ or LC‐MS.^35^, ^36^, ^37^ Concentrations of samples were extrapolated and converted to ng mL^‐1^ (for plasma) or normalised to the wet weight of the tissues (ng g^‐1^ for brain tissues).

### 2.6 Statistical analysis

A two‐way ANOVA was used to analyse the effects of prenatal stress and acute swim stress within each sex. Statistics were carried out using prism, 6.0 (GraphPad Software Inc., San Diego, CA, USA). The results of the two‐way ANOVA are reported above each graph, and individual data points for each experimental group are overlaid on the bar graphs representing the group mean ± SEM. Post‐hoc testing using Fisher's least significance difference test was carried ou. *P* < 0.05 was considered statistically significant. Post‐hoc test results are also reported. To further investigate the presence of any sex differences, three‐way ANOVAs (with sex, acute stress and prenatal stress as main factors) were also carried out (see Supporting information, Table S3).

## 3 RESULTS

### 3.1 Corticosterone

**Figure 3:**
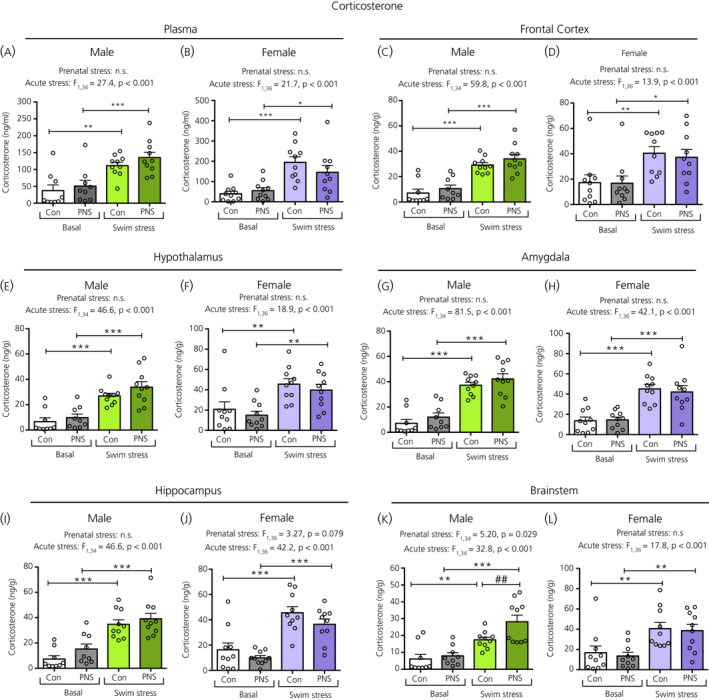
Corticosterone concentrations in the plasma and brain regions of male and female control (Con) and prenatally stressed (PNS) offspring. Data were analysed by two‐way ANOVA with *F* values and *P* values reported above each graph. A significant main effect of acute stress was observed in all regions investigated, for both males and females. All of the swim‐stress exposed groups had significantly greater corticosterone concentrations in the plasma and in the brain regions analysed compared to basal groups (*P < 0.05, **P < 0.01, ***P < 0.001). There was a main effect of prenatal stress observed only in the male brainstem (K), where corticosterone concentrations were significantly greater in male PNS swim‐stressed rats compared to male control swim‐stressed rats (##*P* < 0.01). There were no significant acute stress × prenatal stress interactions in the plasma or in any of the brain regions investigated. Note the difference in the scale of the *y* axes between the sexes. NS, not significant; n = 9 or 10 rats per group

There was a main effect of acute stress on plasma corticosterone concentrations, with significantly greater levels in both control and PNS groups following swim stress in males (Figure 3A) and females (Figure 3B). There was no main effect of prenatal stress on plasma corticosterone concentrations in either sex under basal conditions or following acute swim stress (Figure 3A,B).

A main effect of acute stress was also observed in all brain regions examined, with significantly greater corticosterone concentrations in the control and PNS rats following acute swim stress in both sexes (Figure 3C‐L). An additional main effect of prenatal stress exposure was detected for the brainstem in the male offspring, with post‐hoc testing indicating significantly greater corticosterone concentrations following swim stress in the PNS males compared to the control males (Figure 3K). There were no significant differences in corticosterone concentrations between the control and PNS groups for any of the other brain regions analysed.

### 3.2 DOC

**Figure 4:**
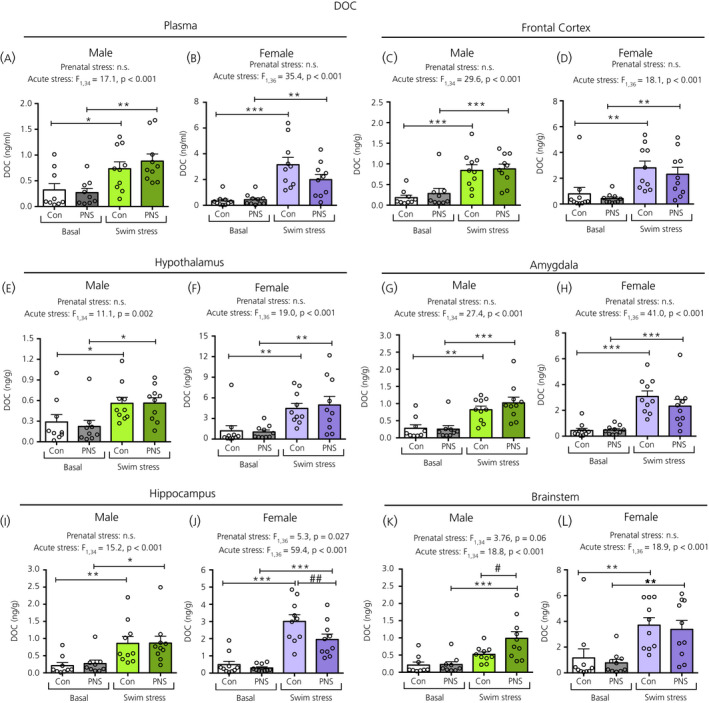
Deoxycorticosterone (DOC) concentrations in the plasma and brain regions of male and female control (Con) and prenatally stressed (PNS) offspring. A significant main effect of acute stress was observed in the plasma and all brain regions investigated. All swim‐stressed groups had significantly greater DOC concentrations compared to basal groups (*P < 0.05, **P < 0.01, ***P < 0.001), except for the male brainstem (K), where only PNS groups showed significant increases in DOC concentrations with swim stress. A main effect of prenatal stress was detected for the male brainstem (K) and female hippocampus (J). Although PNS swim‐stressed males had greater brainstem DOC concentrations compared to male control swim‐stressed rats (##*P* < 0.01), hippocampal DOC concentrations in female swim‐stressed rats were lower compared to female control swim‐stressed rats (#*P* < 0.05). There were no significant acute stress × prenatal stress interactions in the plasma or in any of the brain regions investigated. Note the difference in the scale of the *y* axes between the sexes. NS, not significant. n = 9 or 10 rats per group

There was a main effect of acute stress on plasma DOC concentrations, with significantly greater levels in the swim‐stressed groups compared to the basal groups in both the control and PNS groups, in males (Figure 4A) and females (Figure 4B). There were no effects of prenatal stress on circulating DOC concentrations under basal conditions or in response to acute stress.

A main effect of acute stress was observed for DOC in all brain regions. Rats exposed to swim stress had significantly greater DOC concentrations than non‐swim‐stressed rats, regardless of prenatal stress status in the frontal cortex, hypothalamus, amygdala and hippocampus in males and in all brain regions in females (Figure 4). In the male brainstem, an additional main effect of prenatal stress was observed, with the PNS group having significantly greater swim stress‐induced DOC concentrations compared to the control swim‐stressed group (Figure 4K). An effect of prenatal stress was also observed in the female hippocampus (Figure 4J), where PNS swim‐stressed females had significantly lower DOC concentrations compared to the swim‐stressed control group. There were no differences in DOC concentrations between control and PNS animals in any of the other brain regions examined (Figure 4).

### 3.3 DHDOC and THDOC

**Figure 5:**
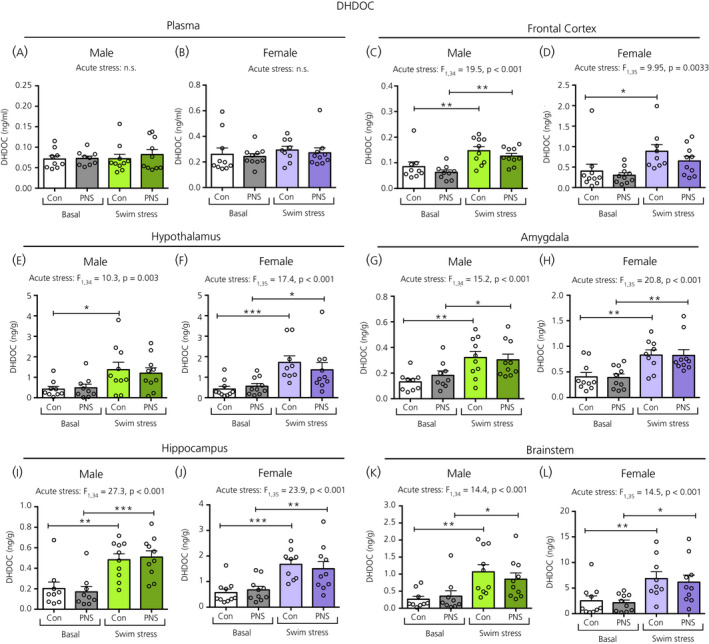
Dihydrodeoxycorticosterone (DHDOC) concentrations in the plasma and brain regions of male and female control (Con) and prenatally stressed (PNS) offspring. There were no significant differences in plasma DHDOC concentrations between the four treatment groups in either males (A) or females (B). Significant main effects of acute stress were observed in all brain regions investigated, in both sexes. Post‐hoc testing revealed that, in most brain regions, swim‐stressed rats had greater DOC concentrations than basal rats (*P < 0.05, **P < 0.01, ***P < 0.001), except for in the female frontal cortex (D) and in the male hypothalamus (E), where swim stress did not result in differences in the PNS groups. There were no significant main effects of prenatal stress, nor any acute stress × prenatal stress interactions in the plasma or in any of the brain regions investigated. Note the difference in the scale of the *y* axes between the sexes. NS, not significant. n = 9 or 10 rats per group

There was no effect of acute stress nor prenatal stress on plasma DHDOC concentrations in either males (Figure 5A) or females (Figure 5B). A main effect of acute stress was observed in all brain regions; however, there was no effect of prenatal stress for any of the brain regions (Figure 5C‐L). Post‐hoc testing revealed that, in most brain areas, both control and PNS rats had significantly greater DHDOC concentrations in the swim stress exposed groups than in the respective basal groups, except for in the frontal cortex of the females (Figure 5D) and in the hypothalamus of the males (Figure 5E), where the stress‐induced increase was significant only in the control groups.

**Figure 6:**
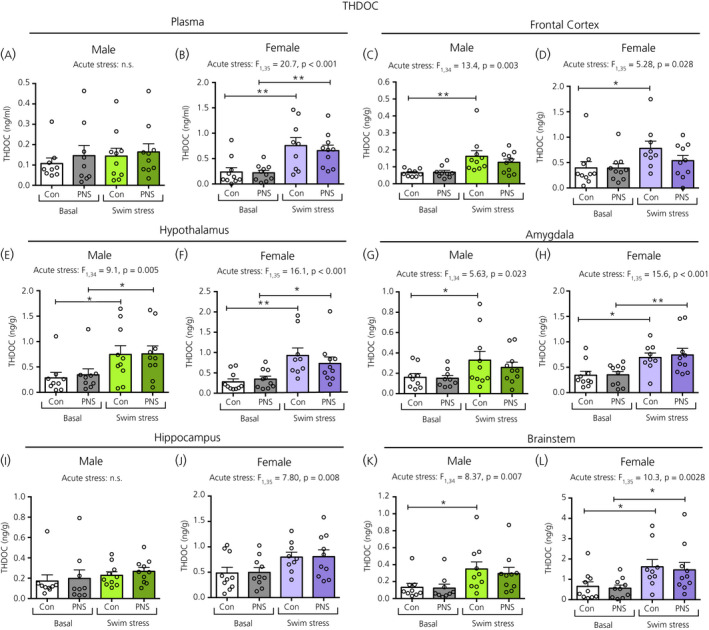
Tetrahydrocorticosterone (THDOC) concentrations in the plasma and brain regions of male and female control (Con) and prenatally stressed (PNS) offspring. A significant main effect of acute stress on plasma THDOC was observed in females, but not in males. A significant main effect of acute stress was also observed in all brain regions, except for the male hippocampus (I). Post‐hoc testing revealed that, although both controls and PNS groups had greater THDOC concentrations following swim stress in the male (E) and female (F) hypothalamus, female amygdala (H) and brainstem (L), the increase was only significant in the control groups in the male and female frontal cortex (C and D) and male amygdala (G) and brainstem (K). There were no significant main effects of prenatal stress, nor any acute stress × prenatal stress interactions in the plasma or in any of the brain regions investigated. Note the difference in the scale of the *y* axes between the sexes. Post‐hoc tests: *P < 0.05, **P < 0.01. NS, not significant. n = 9 or 10 rats per group

For plasma THDOC concentrations, a main effect of acute stress was observed only in females, where plasma THDOC concentrations were significantly increased following swim stress in both the female control and PNS groups (Figure 6B), however they were not different in males (Figure 6A). A main effect of acute stress, but not prenatal stress, was observed in all brain regions, except for in the male hippocampus (Figure 6I). In the hypothalamus of males and females (Figure 6E, F) and the amygdala (Figure 6H) and brainstem (Figure 6L) of female rats, both control and PNS groups showed significantly greater THDOC concentrations following swim stress compared to the basal groups. However, in the frontal cortex of both sexes (Figure 6C, D) and in the amygdala (Figure 6G) and brainstem (Figure 6K) of the male rats, significantly greater THDOC concentrations following swim stress were observed only in the control groups, and not in the PNS rats.

### 3.4 Progesterone, DHP and allopregnanolone

**Figure 7:**
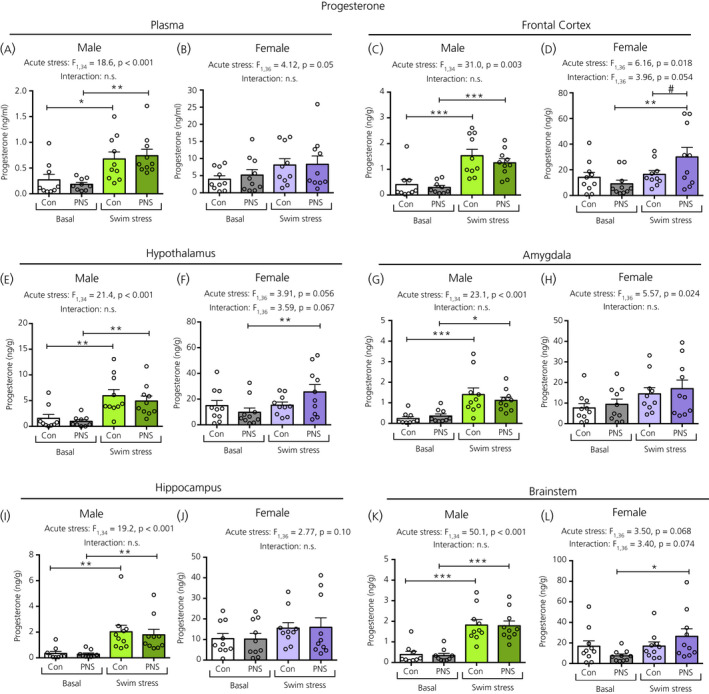
Progesterone concentrations in the plasma and brain regions of male and female control (Con) and prenatally stressed (PNS) offspring. In males, a significant main effect of acute stress was observed in progesterone concentrations in the plasma and in each of the five brain regions, with both control and PNS swim‐stressed males having greater progesterone concentrations compared to the basal groups (post‐hoc tests: *P < 0.05, **P < 0.01, ***P < 0.001). In females, although the two‐way ANOVA revealed a significant main effect of acute stress for the plasma and several brain regions, post‐hoc testing revealed that pairwise comparisons were not significant for either control or PNS groups in the plasma (B), amygdala (G) or hippocampus (J). In the female frontal cortex (D), hypothalamus (F) and brainstem (L), only the PNS swim‐stressed groups showed significantly greater concentrations compared to the basal groups. Additionally, in the frontal cortex of females (D), an acute stress × prenatal stress interaction was detected, and PNS swim‐stressed rats had greater progesterone concentrations compared to control swim‐stressed rats (#*P* < 0.05). No main effect of prenatal stress was observed in the plasma or in any of the five brain regions. Note the difference in the scale of the y axes between the sexes. NS, not significant. n = 9 or 10 rats per group

**Figure 8:**
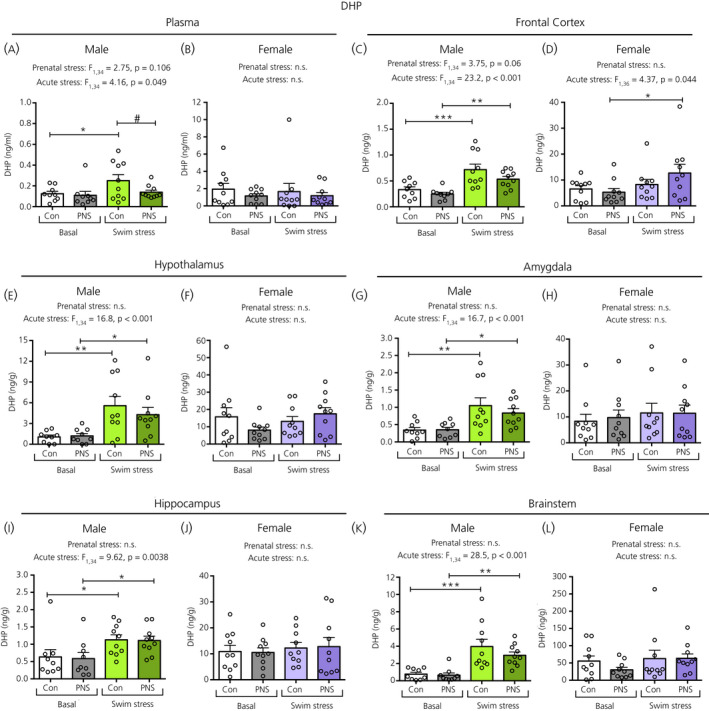
Dihydroprogesterone (DHP) concentrations in the plasma and brain regions of male and female control (Con) and prenatally stressed (PNS) offspring. In the plasma from males (A), a significant main effect of acute stress was observed where control male swim‐stressed rats had greater plasma DHP concentrations than the control basal group (*P < 0.05) and also the PNS swim‐stressed group (#*P* < 0.05). In the brain, significant main effects of acute stress were also observed in males, with swim‐stressed rats having significantly greater DHP concentrations compared to basal groups in both control and PNS conditions (*P < 0.05, **P < 0.01, ***P < 0.001). In females, a main effect of acute stress was only observed in the frontal cortex (H), where the PNS, but not the control, swim‐stressed rats had greater concentrations of DHP compared to their respective basal group (*P < 0.05). There were no significant acute stress × prenatal stress interactions in the plasma or in any of the brain regions investigated. Note the difference in the scale of the *y* axes between the sexes. NS, not significant. n = 9 or 10 rats per group

**Figure 9:**
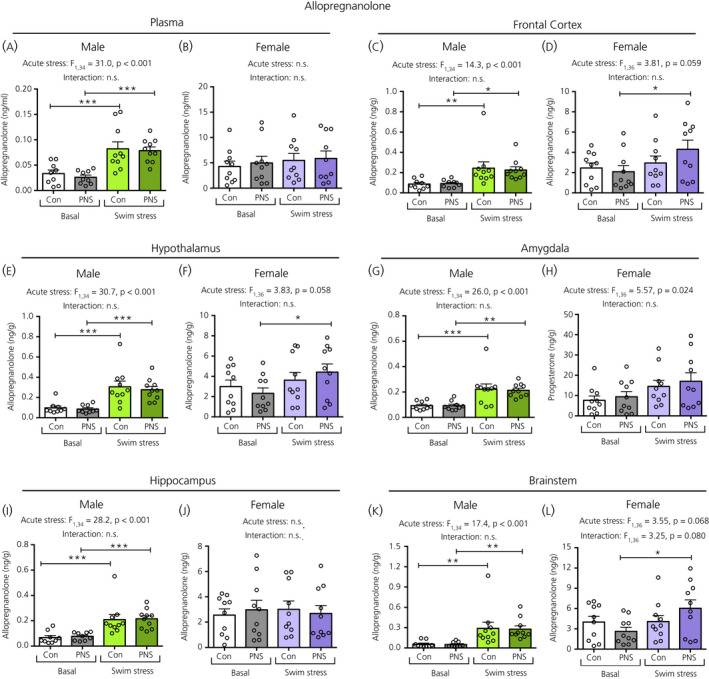
Allopregnanolone concentrations in the plasma and brain regions of male and female control (Con) and prenatally stressed (PNS) offspring. In males, a significant main effect of acute stress was observed for the plasma and all brain regions, where the swim‐stressed rats had greater concentrations of allopregnanolone in the circulation and brain compared to the basal groups, under both control and PNS conditions (*P < 0.05, **P < 0.01, ***P < 0.001). No main effects of prenatal stress were observed in the male plasma or in any of the brain regions. In females, there was no effect of prenatal stress or acute stress on plasma allopregnanolone concentrations. In the brain, no effect of prenatal stress was observed. A main effect of acute stress was observed in the frontal cortex (D), hypothalamus (F), amygdala (H) and brainstem (L) of the females; however, post‐hoc testing revealed significant group differences only in the frontal cortex (D) and hypothalamus (F), where PNS, but not control, swim‐stressed rats had greater concentrations of allopregnanolone compared to their respective basal group (*P < 0.05). Note the difference in the scale of the *y* axes between the sexes. NS, not significant. n = 9 or 10 rats per group

In males, there was a significant main effect of acute stress on plasma progesterone (Figure 7A), DHP (Figure 8A) and allopregnanolone (Figure 9A) concentrations. Post‐hoc testing revealed that, in males, both the control and PNS swim‐stressed groups had significantly greater plasma progesterone (Figure 7A) and allopregnanolone (Figure 8A) concentrations compared to the basal groups. However, for plasma DHP, acute swim stress resulted in greater DHP concentrations only in the control and not in the PNS group (Figure 8A), such that the male controls exposed to swim stress had significantly greater concentrations of plasma DHP compared to male PNS swim‐stress rats (Figure 8A). In females, although a main effect of acute stress was observed for plasma progesterone (Figure 7B), post‐hoc testing indicated that there were no significant differences in circulating progesterone (Figure 7B), DHP (Figure 8B) and allopregnanolone (Figure 9B) concentrations between the four groups.

In males, a main effect of acute stress was observed for all brain regions with swim stress resulting in greater concentrations of progesterone, DHP and allopregnanolone in both control and PNS animals compared to their respective basal groups (Figures 7, 8 and 9). Tissue concentrations of progesterone, DHP and allopregnanolone did not differ significantly between PNS rats and control rats, both under basal conditions and after acute swim stress, in any of the five brain regions investigated (Figures 7, 8, 9).

In females, there were main effects of acute stress in most brain regions for progesterone (Figure 7), although only in the frontal cortex for DHP (Figure 8D), and the frontal cortex (Figure 9D), hypothalamus (Figure 9F), amygdala (Figure 9H) and brainstem (Figure 9L) for allopregnanolone. There were no main effects of prenatal stress, except for on progesterone concentrations in the frontal cortex (Figure 7D) and allopregnanolone concentrations in the brainstem (Figure 9L). However, post‐hoc testing revealed that swim stress had no significant effect on levels of progesterone, DHP or allopregnanolone in any of the brain regions studied in the control groups, whereas there were instances where the PNS swim‐stressed groups showed significantly greater steroid concentrations than the PNS basal group. Specifically, in the frontal cortex, progesterone (Figure 7D), DHP (Figure 8D) and allopregnanolone (Figure 9D) were significantly greater in the PNS groups following swim stress compared to the basal PNS groups and progesterone was significantly greater in the PNS swim‐stressed group than in the control swim‐stressed group (Figure 7D). In the hypothalamus and brainstem, progesterone (Figure 7F and L) and allopregnanolone (Figure 9F and L) levels were also significantly increased by swim stress in the PNS groups, but not in the control groups.

### 3.5 Pregnenolone

**Figure 10:**
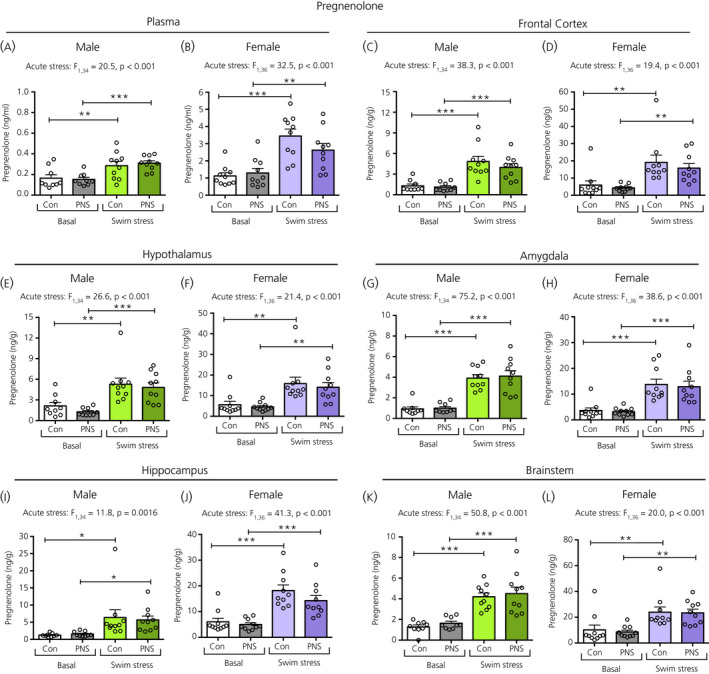
Pregnenolone concentrations in the plasma and brain regions of male and female control (Con) and prenatally stressed (PNS) offspring. A significant main effect of acute stress was observed in all regions investigated, for both males and females. There were no significant main effects of prenatal stress, nor any acute stress × prenatal stress interactions in the plasma or in any of the brain regions investigated. Pregnenolone concentrations in the plasma and each of the brain regions examined were significantly greater in the male and female control and PNS groups exposed to swim stress compared to the basal groups (post‐hoc tests: *P < 0.05, **P < 0.01, ***P < 0.001). Note the difference in the scale of the *y* axes between the sexes. NS, not significant. n = 9 or 10 rats per group

There was a significant main effect of acute stress on pregnenolone concentrations in the plasma and each of the brain regions examined. In both sexes, pregnenolone concentrations were significantly greater in the swim‐stressed groups compared to the respective basal groups, regardless of prenatal stress status (Figure 10). There were no significant differences in pregnenolone concentrations between control and PNS offspring, either under basal conditions or following acute swim stress (Figure 10).

### 3.6 Testosterone

**Figure 11:**
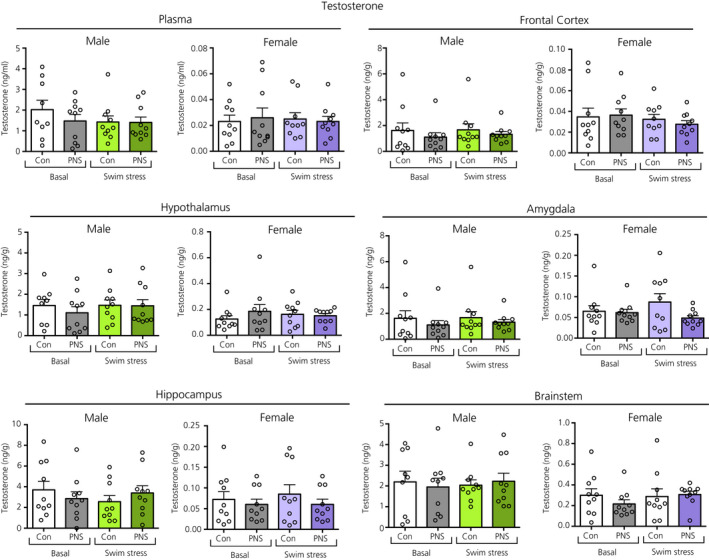
Testosterone concentrations in the plasma and brain regions of male and female control (Con) and prenatally stressed (PNS) offspring. There was no significant effect of prenatal stress or acute swim stress on circulating or central testosterone concentrations in either males or females. n = 9 or 10 rats per group. Note the difference in the scale of the *y* axes between the sexes

There were no main effects of acute stress, prenatal stress, nor any interaction effect on circulating or central testosterone concentrations, in either males or females (Figure 11).

### 3.7 Sex differences

Three‐way ANOVA revealed significant main effects of sex on neuroactive steroid concentrations in the plasma and brain for virtually all samples examined (see Supporting information, Table S3). For corticosterone, DOC, DHDOC, THDOC, progesterone, DHP, allopregnanolone and pregnenolone, concentrations were typically greater in females compared to males, under both basal and stress conditions, regardless of prenatal experience (Figures 3, 4, 5, 6, 7, 8, 9, 10; see also Supporting information, Table S3). By contrast, circulating and central testosterone concentrations were significantly greater in males compared to females, independent of prenatal or acute stress exposure (Figure 11; see also Supporting information, Table S3).

## 4 DISCUSSION

**Figure 12:**
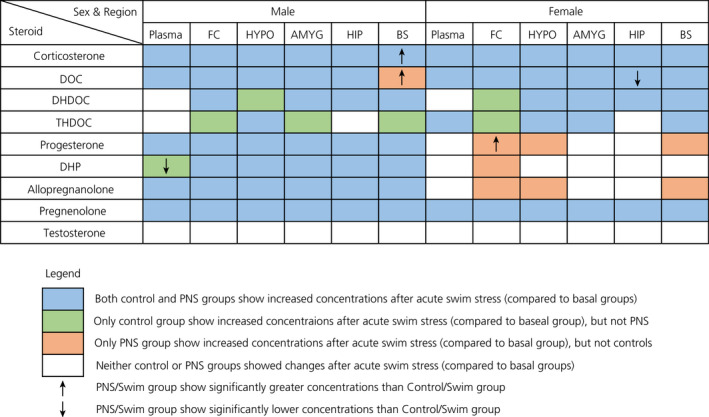
Summary of the main differences in neuroactive steroid production observed between control and prenatally stressed (PNS) groups in the present study. DOC, deoxycorticosterone; DHDOC, dihydrodeoxycorticosterone; THDOC, tetrahydrocorticosterone; DHP, dihydroprogesterone; FC, frontal cortex; HYPO, hypothalamus; AMYG, amygdala; HIP, hippocampus; BS, brainstem; PNS, prenatally stressed

The present study aimed to determine whether the steroidal profile of prenatally stressed offspring differed from that of control offspring, both under basal conditions and following acute swim stress. Overall, there were no differences between PNS and control offspring with respect to basal levels of neuroactive steroids in either the plasma or the brain. Following acute swim stress, concentrations of most of the neuroactive steroids examined increased in PNS offspring in a manner largely similar to the control offspring; however, there were a few exceptions (Figure 12). In particular, swim stress failed to evoke a significant increase in the concentrations of the positive GABA_A_ receptor (GABA_A_R) modulatory steroids, DHDOC and THDOC, in discrete brain regions in PNS offspring of both sexes, whereas it did in controls. In female rats, swim stress typically increased concentrations of progesterone and its metabolites in specific brain regions only in the PNS, and not in the control females.

Here, PNS offspring did not exhibit corticosterone hypersecretion following swim stress (at least at the 30 minute time point studied), in contrast to our findings using acute restraint or immune challenge.^22^, ^23^ However, glucocorticoid concentrations were greater in the brainstem of male PNS swim‐stressed rats compared to the control swim‐stressed rats. The reasons for this are unclear; however, it may relate to the nature of the stressor used in this study as different stress paradigms evoke different neuroendocrine responses as a result of differences in signalling pathways and the subjective appraisal of the stressor.^38^ The neural pathways activated by acute swim stress, which is a combined physical and psychogenic stressor, are complex and evidently involve inputs from brainstem noradrenergic pathways^39^ and serotonergic pathways,^40^ as well as from several limbic brain structures.^41^ This is in contrast with stressors such as IL‐1β administration used in our previous studies, which activates a noradrenergic pathway from brainstem A2 nucleus tractus solitarius neurones that project directly to CRH neurones in the paraventricular nucleus.^42^, ^43^, ^44^ Moreover, because swim stress can be considered forced exercise, there may be additional metabolic consequences,^45^ and/or individual variations in behavioural coping styles ^46^ that further complicate the final neuroendocrine output.

It is also worth noting that the single time point of sample collection here represents a momentary glimpse of the steroid profile in the plasma and brain 30 minutes after the onset of an acute stressor, and thus does not provide information about the time‐course of the stress response. It is possible that the stress response has different temporal dynamics in the PNS offspring; for example, a longer duration for the resolution of the corticosterone response, as described previously,^23^ which we did not assess in the present study. Lastly, although circulatory corticosterone concentrations did not differ between PNS and control offspring, access to the targets in the brain depends on levels and binding capacity of their carrier proteins (eg, corticosteroid binding globulin) in the circulation. Concentrations of these steroids in the brain, rather than in the periphery, may therefore better reflect whether PNS offspring are subject to different central steroid regulatory processes.

Here, compared to control offspring, acute swim stress resulted in greater concentrations of corticosterone and DOC concentrations in the brainstem of the male PNS offspring, whereas, in the female PNS offspring, lower DOC concentrations were observed in the hippocampus. These changes in glucocorticoid concentrations in the brain may result from a different rate and degree of glucocorticoid uptake from the bloodstream, different concentrations and binding affinity of CBG, or altered action of 11β‐hydrogenase enzymes in the brain between male and female control and PNS offspring. Because DOC acts on both GR and MR, these observations may indicate glucocorticoid signalling is differentially affected in the male and female PNS offspring.^47^, ^48^ The hippocampus plays an important role in the negative‐feedback control of the HPA axis, where decreased glucocorticoid action may result in impaired negative‐feedback control.^49^ Although hippocampal GR mRNA expression is similar in male control and PNS offspring, levels are significantly lower in the CA2 region of PNS females compared to control females.^23^ Moreover, hippocampal MR mRNA expression is markedly lower in PNS offspring across all subfields in both sexes.^23^ This may further exacerbate the effects of the lower hippocampal DOC concentrations in the female PNS offspring and could contribute to impaired glucocorticoid negative‐feedback, which may explain sex differences in the amplitude and resolution of the stress response previously reported.^4^, ^23^

With respect to the positive GABA_A_R modulatory steroids, allopregnanolone levels were not lower in PNS offspring compared to controls, in either sex, in contrast to our hypothesis based on our previous studies.^22^ Indeed, swim stress resulted in greater allopregnanolone concentrations in the frontal cortex, hypothalamus and brainstem of the PNS females, although not the control females. Although studies have shown that allopregnanolone administration can rescue anxiety and depression‐like behaviour, as well as ameliorate HPA axis hyperactivity in prenatally stressed animals,^22^, ^24^ it is likely that this effect of exogenous steroids occurs independent of deficits in endogenous levels. Alternatively, it may be that, rather than altered levels of GABA_A_R modulatory steroids, PNS offspring exhibit different GABA_A_R receptor expression or activation patterns compared to controls,^50^ as has been reported for other models of prenatal stress.^51^ Indeed, we have evidence that indicate GABA_A_R are down‐regulated in the hippocampus and amygdala of prenatally stressed offspring in this model (T.J. Phillips, Y. Sze & P. J. Brunton, unpublished observations). Concentrations of the allopregnanolone precursor, DHP, in the brain also did not differ between male control and PNS animals in the present study, despite a deficit in circulating DHP concentrations in the male PNS offspring. Although it is unknown whether lower plasma DHP concentrations has implications on, for example, peripheral metabolism, this observation may result from decreased hepatic 5α‐reductase 1 gene expression that we previously reported.^52^

Although the central allopregnanolone response to stress does not appear to be compromised in the PNS offspring, there is evidence for modest deficits in DHDOC and THDOC production in both the male and female PNS offspring (Figure 12). Because DHDOC and THDOC also act as positive allosteric modulators on GABA_A_R receptor,^48^ this observation suggests that inhibitory signalling in PNS offspring may be affected. Although this again would depend on GABA_A_R receptor dynamics, it is of interest given that dysfunctional GABAergic signalling is implicated in the pathophysiology of psychiatric disorders, for which prenatal stress is a risk factor.^50^, ^53^ Despite a role for THDOC in regulating HPA axis responses to stress being well established,^14^, ^54^ further studies are necessary to determine whether the differences observed in the present study, which are in relatively small, would have any significant physiological impact for HPA axis function. Nonetheless, a role for THDOC in stress‐related disorders warrants further investigation because there is a relative paucity of studies available, despite the observation that THDOC concentrations are elevated following acute stress,^3^ and that its administration can rescue deficits associated with prenatal stress.^31^ It is not known whether THDOC or allopregnanolone play complementary or compensatory roles as allosteric mediators of the stress response, although there is evidence that THDOC and allopregnanolone can alter synaptic inhibition differently. In vivo electrophysiology experiments demonstrate the potency of THDOC to be greater than that of allopregnanolone with certain GABA_A_ receptor subunit combinations, indicating that THDOC may be more effective than allopregnanolone in inducing an inhibitory tonic current.^55^, ^56^, ^57^ Moreover, it is not clear whether the increased levels of allopregnanolone, as observed here in the PNS females following acute stress, compensate for any deficits in central DHDOC/THDOC production, although, if this were the case, it may help explain sex differences in anxiety‐like behaviour, where PNS males, but not females, are affected.^23^ Indeed, greater allopregnanolone (and its precursors) responses in PNS females could be an adaptive response helping these animals to better cope with stress encountered in adulthood, in line with the match‐mismatch hypothesis of early‐life programming.^58^

Consistent with our previous findings,^4^ there were marked sex differences in the concentrations of steroids measured in almost all brain regions; where corticosterone, DOC, DHDOC, THDOC, progesterone, DHP, allopregnanolone and pregnenolone concentrations were greater in females, whereas testosterone concentrations were markedly greater in males. These sex differences likely reflect differences in the levels of precursors and/or the activity of the steroidogenic converting enzymes. For example, higher levels of progesterone in females compared to males likely contribute to the greater levels of several of the downstream steroids for which progesterone is the precursor (Figure 1). Moreover, the central expression of key enzymes involved in neuroactive steroid synthesis is sexually dimorphic,^59^ which may contribute to sex differences in neuroactive steroid levels in the brain. The impact of these sex differences on function are unclear; however, given the well established roles of neuroactive steroids in modulating stress responses and mood,^16^, ^26^, ^27^ differences in neuroactive steroid concentrations in the brain between males and female may contribute to the sex differences in HPA axis regulation and susceptibility to psychiatric disorders.^29^, ^60^, ^61^, ^62^

Additionally, although sex differences in central and peripheral allopregnanolone levels have been described previously, both under basal conditions and following acute stress,^29^ this is the first study to report sex differences in the THDOC response to stress. In the main, females had greater basal and stress‐induced THDOC concentrations not only in the brain, but also in the plasma, a finding not seen in males. It is therefore likely that, similar to observations for allopregnanolone,^63^, ^64^ THDOC sensitivity may be different for males and females. Indeed, differences in the maximal GABA_A_ receptor potentiation of THDOC between male and female rats have been reported previously.^65^

Although it is not possible to determine the origin of the steroids measured in the brain in response to stress in the present study, both changes in secretion by peripheral steroidogenic organs, (such as the adrenal glands and gonads) for some steroids, as well as local synthesis in the brain for others, are expected to contribute. For example, DOC is synthesised from progesterone predominantly in the adrenal cortex and is secreted with corticosterone and progesterone following stress exposure.^2^, ^48^ Moreover, we have previously reported that central concentrations of corticosterone, progesterone and DOC following acute swim stress are positively correlated with circulating levels,^4^ indicating that changes in the brain likely reflect changes in production in the periphery. On the other hand, we have also demonstrated that DHP, DHDOC and allopregnanolone concentrations in the brain following acute swim stress do not correlate with circulating levels,^4^ supporting the concept of local synthesis of these neuroactive steroids in the brain in response to stress. Indeed, in the present study, although acute stress failed to increase circulating DHDOC concentrations in both sexes and THDOC in males, central DHDOC and THDOC concentrations were increased in these animals. Similarly, allopregnanolone concentrations in the brain were elevated after stress in females, despite no corresponding increase in plasma allopregnanolone concentrations. Taken together, these data support the concept of production of these steroids within the brain, through the actions of 5α‐reductase and 3α‐hydroxysteroid dehydrogenase.^7^ Indeed, acute swim stress up‐regulates central 5α‐reductase (the rate‐limiting enzyme) expression, at least in naïve male rats.^66^ which would be expected to promote DHDOC/DHP production and subsequently THDOC/allopregnanolone synthesis. Although PNS affects baseline 5α‐reductase gene expression in a sex‐ and region‐dependent manner,^22^ it remains to be established whether prior PNS exposure and/or sex alters the stress‐induced increase in 5α‐reductase in the brain, which could potentially explain the different steroid responses to stress in male and female PNS animals.

In summary, the present study provides a comprehensive examination of nine neuroactive steroids in five brain regions in male and female control and prenatally stressed (PNS) rats. We conclude that, under basal conditions, there are no major differences in circulating and central neuroactive steroid concentrations between control and PNS offspring. However, there are modest differences in how PNS and control offspring respond to acute swim stress, particularly in terms of DHDOC and THDOC production in the brain, which may indicate deficits in GABAergic signalling in PNS offspring, and this could contribute to differences in HPA axis responses to stress and altered stress‐related behaviours in PNS rats.^22^, ^23^ Nevertheless, it is worthwhile highlighting again that plasma corticosterone concentrations were not significantly different between control and PNS offspring at the time point studied; therefore, it remains unclear whether more striking differences in neuroactive steroid concentrations related to HPA axis regulation may emerge at different time points, or in response to different stressors. On the other hand, the lack of robust differences may reflect the complicated interaction between prenatal programming and the acute stress response, and also underlines the complex nature of steroid action in the brain, for example the finding that THDOC is both necessary for mounting and for terminating HPA axis responses to stress.^14^ We propose a more integrative approach to investigating the contribution of steroid action following both prenatal and acute stress, aiming to establish how various allostatic mediators may interact to regulate stress responses. The THDOC response to stress is likely to play an adaptive role; hence, differences in production and/or action in the brain may contribute to sex differences in HPA axis function and in the dysregulation induced by prenatal stress and warrants further investigation.

## CONFLICT OF INTERESTS

The authors declare that they have no conflicts of interest.

## AUTHOR CONTRIBUTIONS

**Sze Ying:** Conceptualisation; Data curation; Formal analysis; Investigation; Methodology; Validation; Visualisation; Writing – original draft; Writing – review & editing. **Paula J Brunton:** Conceptualisation; Funding acquisition; Investigation; Methodology; Project administration; Supervision; Writing – original draft; Writing – review & editing.

### Peer Review

The peer review history for this article is available at https://publons.com/publon/10.1111/jne.12916.

## Supporting information

Supplementary Material

## Data Availability

The data that support the findings of this study are available from the corresponding author upon reasonable request.

## References

[1] McEwen BS . Protective and damaging effects of stress mediators: central role of the brain. Dialogues Clin Neurosci. 2006;8:367‐381. 17290796 10.31887/DCNS.2006.8.4/bmcewen PMC3181832

[2] Hueston CM , Deak T . On the time course, generality, and regulation of plasma progesterone release in male rats by stress exposure. Endocrinology. 2014;155:3527‐3537. 24926824 10.1210/en.2014-1060

[3] Purdy RH , Morrow AL , Moore PH Jr , Paul SM . Stress‐induced elevations of gamma‐aminobutyric acid type A receptor‐active steroids in the rat brain. Proc Natl Acad Sci U S A. 1991;88:4553‐4557. 1852011 10.1073/pnas.88.10.4553 PMC51699

[4] Sze Y , Gill AC , Brunton PJ . Sex‐dependent changes in neuroactive steroid concentrations in the rat brain following acute swim stress. J Neuroendocrinol. 2018;30:e12644. 30194779 10.1111/jne.12644 PMC6221110

[5] Melcangi RC , Garcia‐Segura LM , Mensah‐Nyagan AG . Neuroactive steroids: state of the art and new perspectives. Cell Mol Life Sci. 2008;65:777‐797. 18038216 10.1007/s00018-007-7403-5 PMC11131680

[6] Baulieu EE . Neurosteroids: of the nervous system, by the nervous system, for the nervous system. Recent Prog Horm Res. 1997;52:1‐32. 9238846

[7] Agis‐Balboa RC , Pinna G , Zhubi A , et al. Characterization of brain neurons that express enzymes mediating neurosteroid biosynthesis. Proc Natl Acad Sci USA. 2006;103:14602‐14607. 16984997 10.1073/pnas.0606544103 PMC1600006

[8] Giraldi T , Giovannelli P , Di Donato M , Castoria G , Migliaccio A , Auricchio F . Steroid signaling activation and intracellular localization of sex steroid receptors. J Cell Commun Signal. 2010;4:161‐172. 21234121 10.1007/s12079-010-0103-1 PMC2995128

[9] Lambert JJ , Cooper MA , Simmons RD , Weir CJ , Belelli D . Neurosteroids: endogenous allosteric modulators of GABA(A) receptors. Psychoneuroendocrinology. 2009;34(Suppl 1):S48‐58. 19758761 10.1016/j.psyneuen.2009.08.009

[10] Sedlacek M , Korinek M , Petrovic M , et al. Neurosteroid modulation of ionotropic glutamate receptors and excitatory synaptic transmission. Physiol Res. 2008;57(Suppl 3):S49‐57. 10.33549/physiolres.931600 18481915

[11] Paul SM , Purdy RH . Neuroactive steroids. FASEB J. 1992;6:2311‐2322. 1347506

[12] Joels M . Corticosteroids and the brain. J Endocrinol. 2018;238:R121‐R130. 29875162 10.1530/JOE-18-0226

[13] Tasker JG , Herman JP . Mechanisms of rapid glucocorticoid feedback inhibition of the hypothalamic‐pituitary‐adrenal axis. Stress. 2011;14:398‐406. 21663538 10.3109/10253890.2011.586446 PMC4675656

[14] Sarkar J , Wakefield S , MacKenzie G , Moss SJ , Maguire J . Neurosteroidogenesis is required for the physiological response to stress: role of neurosteroid‐sensitive GABAA receptors. J Neurosci. 2011;31:18198‐18210. 22171026 10.1523/JNEUROSCI.2560-11.2011 PMC3272883

[15] Wirth MM . Beyond the HPA axis: progesterone‐derived neuroactive steroids in human stress and emotion. Front Endocrinol. 2011;2:19. 10.3389/fendo.2011.00019 PMC3355912 22649366

[16] Gunn BG , Cunningham L , Mitchell SG , Swinny JD , Lambert JJ , Belelli D . GABAA receptor‐acting neurosteroids: a role in the development and regulation of the stress response. Front Neuroendocrinol. 2015;36:28‐48. 24929099 10.1016/j.yfrne.2014.06.001 PMC4349499

[17] Weinstock M . Prenatal stressors in rodents: effects on behavior. Neurobiol Stress. 2017;6:3‐13. 28229104 10.1016/j.ynstr.2016.08.004 PMC5314420

[18] Van den Bergh BRH , van den Heuvel MI , Lahti M , et al. Prenatal developmental origins of behavior and mental health: the influence of maternal stress in pregnancy. Neurosci Biobehav Rev. 2017; 10.1016/j.neubiorev.2017.07.003. Online ahead of print.. 10.1016/j.neubiorev.2017.07.003 28757456

[19] Maccari S , Krugers HJ , Morley‐Fletcher S , Szyf M , Brunton PJ . The consequences of early‐life adversity: neurobiological, behavioural and epigenetic adaptations. J Neuroendocrinol. 2014;26:707‐723. 25039443 10.1111/jne.12175

[20] Maccari S , Darnaudery M , Morley‐Fletcher S , Zuena AR , Cinque C , Van Reeth O . Prenatal stress and long‐term consequences: implications of glucocorticoid hormones. Neurosci Biobehav Rev. 2003;27:119‐127. 12732228 10.1016/s0149-7634(03)00014-9

[21] Huizink AC , de Rooij SR . Prenatal stress and models explaining risk for psychopathology revisited: Generic vulnerability and divergent pathways. Dev Psychopathol. 2018;30:1041‐1062. 30068410 10.1017/S0954579418000354

[22] Brunton PJ , Donadio MV , Yao ST , et al. 5alpha‐Reduced neurosteroids sex‐dependently reverse central prenatal programming of neuroendocrine stress responses in rats. J Neurosci. 2015;35:666‐677. 25589761 10.1523/JNEUROSCI.5104-13.2015 PMC4293416

[23] Brunton PJ , Russell JA . Prenatal social stress in the rat programmes neuroendocrine and behavioural responses to stress in the adult offspring: sex‐specific effects. J Neuroendocrinol. 2010;22:258‐271. 20136688 10.1111/j.1365-2826.2010.01969.x

[24] Zimmerberg B , Blaskey LG . Prenatal stress effects are partially ameliorated by prenatal administration of the neurosteroid allopregnanolone. Pharmacol Biochem Behav. 1998;59:819‐827. 9586837 10.1016/s0091-3057(97)00540-6

[25] Evans J , Sun Y , McGregor A , Connor B . Allopregnanolone regulates neurogenesis and depressive/anxiety‐like behaviour in a social isolation rodent model of chronic stress. Neuropharmacology. 2012;63:1315‐1326. 22939998 10.1016/j.neuropharm.2012.08.012

[26] Gunn BG , Brown AR , Lambert JJ , Belelli D . Neurosteroids and GABA(A) receptor interactions: a focus on stress. Front Neurosci. 2011;5:131. 22164129 10.3389/fnins.2011.00131 PMC3230140

[27] Brunton PJ . Neuroactive steroids and stress axis regulation: Pregnancy and beyond. J Steroid Biochem Mol Biol. 2016;160:160‐168. 26259885 10.1016/j.jsbmb.2015.08.003

[28] Ordyan NE , Pivina SG . Effects of prenatal stress on the activity of an enzyme involved in neurosteroid synthesis during the "critical period" of sexual differentiation of the brain in male rats. Neurosci Behav Physiol. 2005;35:931‐935. 16270175 10.1007/s11055-005-0148-4

[29] Sze Y , Brunton PJ . Sex, stress and steroids. Eur J Neurosci. 2019;52:2487‐2515. 31705553 10.1111/ejn.14615

[30] Ulrich‐Lai YM , Herman JP . Neural regulation of endocrine and autonomic stress responses. Nat Rev Neurosci. 2009;10:397‐409. 19469025 10.1038/nrn2647 PMC4240627

[31] Patchev VK , Hassan AH , Holsboer DF , Almeida OF . The neurosteroid tetrahydroprogesterone attenuates the endocrine response to stress and exerts glucocorticoid‐like effects on vasopressin gene transcription in the rat hypothalamus. Neuropsychopharmacology. 1996;15:533‐540. 8946427 10.1016/S0893-133X(96)00096-6

[32] Vallée M , Rivera JD , Koob GF , Purdy RH , Fitzgerald RL . Quantification of neurosteroids in rat plasma and brain following swim stress and allopregnanolone administration using negative chemical ionization gas chromatography/mass spectrometry. Anal Biochem. 2000;287:153‐166. 11078595 10.1006/abio.2000.4841

[33] Corpechot C , Collins BE , Carey MP , Tsouros A , Robel P , Fry JP . Brain neurosteroids during the mouse oestrous cycle. Brain Res. 1997;766:276‐280. 9359616 10.1016/s0006-8993(97)00749-x

[34] Meffre D , Pianos A , Liere P , et al. Steroid profiling in brain and plasma of male and pseudopregnant female rats after traumatic brain injury: analysis by gas chromatography/mass spectrometry. Endocrinology. 2007;148:2505‐2517. 17303653 10.1210/en.2006-1678

[35] Caruso D , Pesaresi M , Maschi O , Giatti S , Garcia‐Segura LM , Melcangi RC . Effect of short‐and long‐term gonadectomy on neuroactive steroid levels in the central and peripheral nervous system of male and female rats. J Neuroendocrinol. 2010;22:1137‐1147. 20819120 10.1111/j.1365-2826.2010.02064.x

[36] Tobiansky DJ , Korol AM , Ma C , et al. Testosterone and corticosterone in the mesocorticolimbic system of male rats: effects of gonadectomy and caloric restriction. Endocrinology. 2018;159:450‐464. 29069423 10.1210/en.2017-00704

[37] Park MH , Rehman SU , Kim IS , Choi MS , Yoo HH . Stress‐induced changes of neurosteroid profiles in rat brain and plasma under immobilized condition. J Pharm Biomed Anal. 2017;138:92‐99. 28189891 10.1016/j.jpba.2017.02.007

[38] Pacak K , Palkovits M . Stressor specificity of central neuroendocrine responses: implications for stress‐related disorders. Endocr Rev. 2001;22:502‐548. 11493581 10.1210/edrv.22.4.0436

[39] Douglas AJ , Meddle SL , Toschi N , Bosch OJ , Neumann ID . Reduced activity of the noradrenergic system in the paraventricular nucleus at the end of pregnancy: implications for stress hyporesponsiveness. J Neuroendocrinol. 2005;17:40‐48. 15720474 10.1111/j.1365-2826.2005.01272.x

[40] Roche M , Commons KG , Peoples A , Valentino RJ . Circuitry underlying regulation of the serotonergic system by swim stress. J Neurosci. 2003;23:970‐977. 12574426 10.1523/JNEUROSCI.23-03-00970.2003 PMC6741925

[41] Duncan GE , Knapp DJ , Johnson KB , Breese GR . Functional classification of antidepressants based on antagonism of swim stress‐induced fos‐like immunoreactivity. J Pharmacol Exp Ther. 1996;277:1076‐1089. 8627519

[42] Cunningham ET Jr , Sawchenko PE . Anatomical specificity of noradrenergic inputs to the paraventricular and supraoptic nuclei of the rat hypothalamus. J Comp Neurol. 1988;274:60‐76. 2458397 10.1002/cne.902740107

[43] Sawchenko PE , Li HY , Ericsson A . Circuits and mechanisms governing hypothalamic responses to stress: a tale of two paradigms. Prog Brain Res. 2000;122:61‐78. 10737051 10.1016/s0079-6123(08)62131-7

[44] Ericsson A , Kovacs KJ , Sawchenko PE . A functional anatomical analysis of central pathways subserving the effects of interleukin‐1 on stress‐related neuroendocrine neurons. J Neurosci. 1994;14:897‐913. 8301368 10.1523/JNEUROSCI.14-02-00897.1994 PMC6576823

[45] Abel EL . A further analysis of physiological‐changes in rats in the forced swim test. Physiol Behav. 1994;56:795‐800. 7800751 10.1016/0031-9384(94)90245-3

[46] Koolhaas JM , de Boer SF , Coppens CM , Buwalda B . Neuroendocrinology of coping styles: towards understanding the biology of individual variation. Front Neuroendocrinol. 2010;31:307‐321. 20382177 10.1016/j.yfrne.2010.04.001

[47] Gomez‐Sanchez E , Gomez‐Sanchez CE . The multifaceted mineralocorticoid receptor. Compr Physiol. 2014;4:965‐994. 24944027 10.1002/cphy.c130044 PMC4521600

[48] Reddy DS . Physiological role of adrenal deoxycorticosterone‐derived neuroactive steroids in stress‐sensitive conditions. Neuroscience. 2006;138:911‐920. 16325348 10.1016/j.neuroscience.2005.10.016

[49] Jacobson L , Sapolsky R . The role of the hippocampus in feedback regulation of the hypothalamic‐pituitary‐adrenocortical axis. Endocr Rev. 1991;12:118‐134. 2070776 10.1210/edrv-12-2-118

[50] Fine R , Zhang J , Stevens HE . Prenatal stress and inhibitory neuron systems: implications for neuropsychiatric disorders. Mol Psychiatry. 2014;19:641‐651. 24751963 10.1038/mp.2014.35 PMC4031286

[51] Laloux C , Mairesse J , Van Camp G , et al. Anxiety‐like behaviour and associated neurochemical and endocrinological alterations in male pups exposed to prenatal stress. Psychoneuroendocrinology. 2012;37:1646‐1658. 22444623 10.1016/j.psyneuen.2012.02.010

[52] Brunton PJ , Sullivan KM , Kerrigan D , Russell JA , Seckl JR , Drake AJ . Sex‐specific effects of prenatal stress on glucose homoeostasis and peripheral metabolism in rats. J Endocrinol. 2013;217:161‐173. 23428582 10.1530/JOE-12-0540

[53] Selten M , van Bokhoven H , Nadif KN . Inhibitory control of the excitatory/inhibitory balance in psychiatric disorders. F1000Research. 2018;7:23. 29375819 10.12688/f1000research.12155.1 PMC5760969

[54] Morrow AL , Devaud LL , Purdy RH , Paul SM . Neuroactive steroid modulators of the stress response. Ann N Y Acad Sci. 1995;771:257‐272. 8597405 10.1111/j.1749-6632.1995.tb44687.x

[55] Schwabe K , Gavrilovici C , McIntyre DC , Poulter MO . Neurosteroids exhibit differential effects on mIPSCs recorded from normal and seizure prone rats. J Neurophysiol. 2005;94:2171‐2181. 15928052 10.1152/jn.01233.2004

[56] Rahman M , Zhu D , Lindblad C , et al. GABA‐site antagonism and pentobarbital actions do not depend on the alpha‐subunit type in the recombinant rat GABA receptor. Acta Physiol (Oxf). 2006;187:479‐488. 16866778 10.1111/j.1748-1716.2006.01593.x

[57] Locci A , Pinna G . Neurosteroid biosynthesis down‐regulation and changes in GABAA receptor subunit composition: a biomarker axis in stress‐induced cognitive and emotional impairment. Br J Pharmacol. 2017;174:3226‐3241. 28456011 10.1111/bph.13843 PMC5595768

[58] Santarelli S , Lesuis SL , Wang XD , et al. Evidence supporting the match/mismatch hypothesis of psychiatric disorders. Eur Neuropsychopharmacol. 2014;24:907‐918. 24589292 10.1016/j.euroneuro.2014.02.002

[59] Giatti S , Diviccaro S , Garcia‐Segura LM , Melcangi RC . Sex differences in the brain expression of steroidogenic molecules under basal conditions and after gonadectomy. J Neuroendocrinol. 2019;31:e12736. 31102564 10.1111/jne.12736

[60] Altemus M , Sarvaiya N , Epperson CN . Sex differences in anxiety and depression clinical perspectives. Front Neuroendocrinol. 2014;35:320‐330. 24887405 10.1016/j.yfrne.2014.05.004 PMC4890708

[61] Hillerer KM , Slattery DA , Pletzer B . Neurobiological mechanisms underlying sex‐related differences in stress‐related disorders: Effects of neuroactive steroids on the hippocampus. Front Neuroendocrinol. 2019;55:100796. 31580837 10.1016/j.yfrne.2019.100796 PMC7115954

[62] Maguire J . Neuroactive steroids and GABAergic involvement in the neuroendocrine dysfunction associated with major depressive disorder and postpartum depression. Front Cell Neurosci. 2019;8:83. 10.3389/fncel.2019.00083 PMC6418819 30906252

[63] Kelley MH , Kuroiwa M , Taguchi N , Herson PS . Sex difference in sensitivity to allopregnanolone neuroprotection in mice correlates with effect on spontaneous inhibitory post synaptic currents. Neuropharmacology. 2011;61:724‐729. 21640735 10.1016/j.neuropharm.2011.05.017 PMC3133674

[64] Reddy DS , Castaneda DC , O'Malley BW , Rogawski MA . Anticonvulsant activity of progesterone and neurosteroids in progesterone receptor knockout mice. J Pharmacol Exp Ther. 2004;310:230‐239. 14982969 10.1124/jpet.104.065268

[65] Wilson MA , Biscardi R . Influence of gender and brain region on neurosteroid modulation of GABA responses in rats. Life Sci. 1997;60:1679‐1691. 9129123 10.1016/s0024-3205(97)00110-0

[66] Sanchez P , Torres JM , Gavete P , Ortega E . Effects of swim stress on mRNA and protein levels of steroid 5alpha‐reductase isozymes in prefrontal cortex of adult male rats. Neurochem Int. 2008;52:426‐431. 17826869 10.1016/j.neuint.2007.07.019

