# Supplementary material for: Effects of prenatal stress on neuroactive steroid responses to acute stress in adult male and female rats
Source: J Neuroendocrinol. 2020 Dec 3;33(1):e12916. doi: 10.1111/jne.12916 (PMC7900968; doi:10.1111/jne.12916)
Supplement: Supplementary file 1 — Supplementary Material [file JNE-33-e12916-s001.pdf]

SUPPORTING INFORMATION

Effects of prenatal stress on neuroactive steroid responses to acute stress in adult male and female rats

Ying Sze[1,2] and Paula J. Brunton[1,2,3*]

[1]*Centre for Discovery Brain Sciences; and* [2]*The Roslin Institute, University of Edinburgh, UK;* [3]*Zhejiang University-University of Edinburgh Joint* Institute, *Haining, Zhejiang, P.R. China.*

***Corresponding Author**: Centre for Discovery Brain Sciences, University of Edinburgh, Hugh Robson Building, George Square, Edinburgh, EH8 9XD, UK. Email: p.j.brunton@ed.ac.uk

Running title: Prenatal stress and neuroactive steroids

No. of figures: 4 **No. of tables:** 3

Supplementary Figure 1

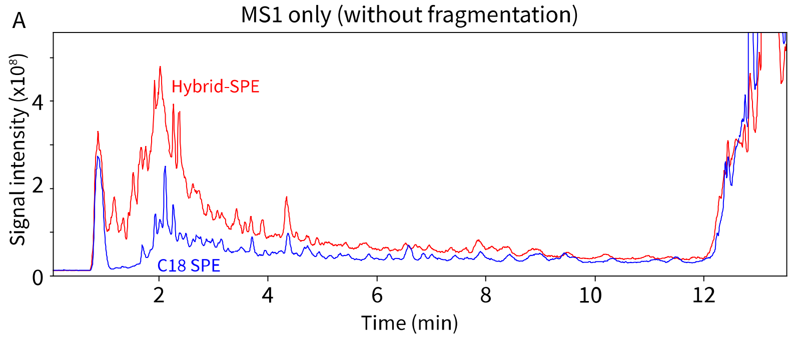

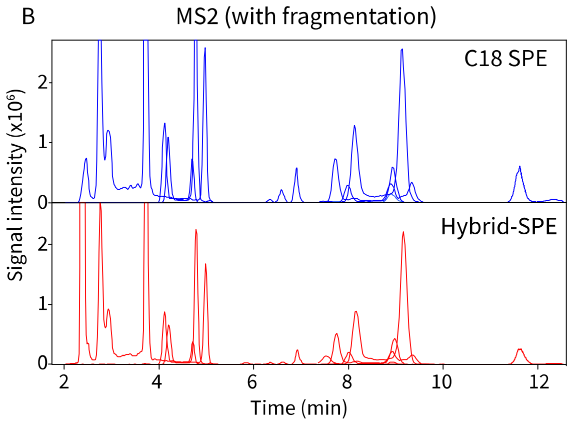

C Area under curve in MS2 mode (Arbitrary units)

	C18 SPE	Hybrid SPE
Corticosterone	4.1×10^7	2.6×10^7
DOC	1.1×10^7	7.1×10^6
DHDOC	1.3×10^6	2.9×10^5
Progesterone	2.5×10^7	2.0×10^7
Progesterone d9	5.5×10^6	4.3×10^6
DHP	3.0×10^6	1.9×10^6
Allopregnanolone	6.7×10^6	3.0×10^6
Testosterone	1.7×10^7	1.0×10^7
Pregnenolone	5.4×10^6	3.9×10^6

Figure S1: Sample clean-up and comparison of different methods of extraction

Sample clean up comparison was carried out using a rat brainstem sample spiked with 0.5 µg of steroid standards. Two methods were compared, C18 method presented in this study, and phospholipid removal using Hybrid-SPE cartridges (Supelco Discovery DSC-18 SPE Cartridge, Sigma, #52602-U, bed weight 100 mg), where samples in methanol/1% FA were loaded through the cartridges as per manufacturer's instructions. Sample clean-up with Hybrid-SPE (in red) showed greater levels of contamination as compared to that of C18-SPE (in blue), evident from the higher total ion intensity in the MS1 (non-fragmentation) mode (A). In the MS2 (fragmentation) mode, C18 SPE method used in this study resulted in a greater signal intensity (B) and area under curve (C) for almost all analytes, indicating better ionisation and fragmentation of analytes.

Supplementary Figure 2

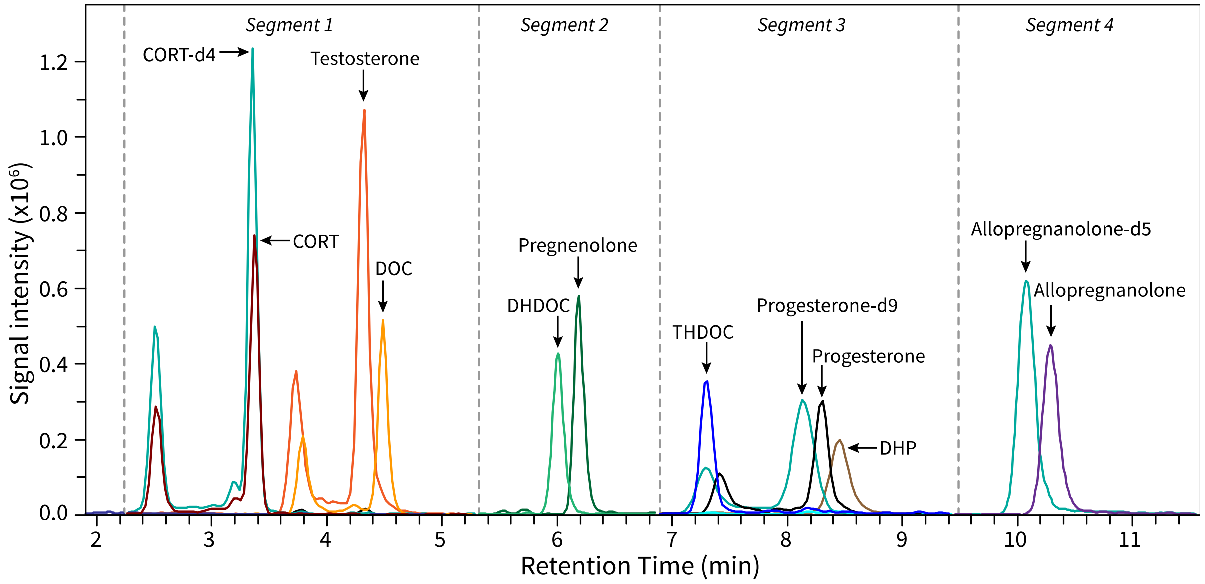

Figure S2: Representative extracted ion chromatogram of steroid analytes (4 ng/ml of standards in 4% BSA).

Girard's T derivatisation resulted in double peaks due to the syn- and anti-configurations of the derivatisation agent. The major peak (the peak with a later retention time) was used for quantification and the ratio between the minor and major peak was always constant. Although the standard calibrants were dissolved in a surrogate matrix of 4% BSA, the inclusion of three internal standards (corticosterone-d4, 3.5 min; progesterone-d9, 8.2 min; allopregnanolone-d5, 10.5 min) helps mitigate any potential matrix effects that may be present at various points across the elution profile in brain tissue.

Abbreviations: CORT, corticosterone; CORT-d4, corticosterone-d4; DOC, deoxycorticosterone; DHDOC, dihydrodeoxycorticosterone; THDOC, tetrahydrodeoxycorticosterone; DHP, dihydroprogesterone

Supplementary Figure 3

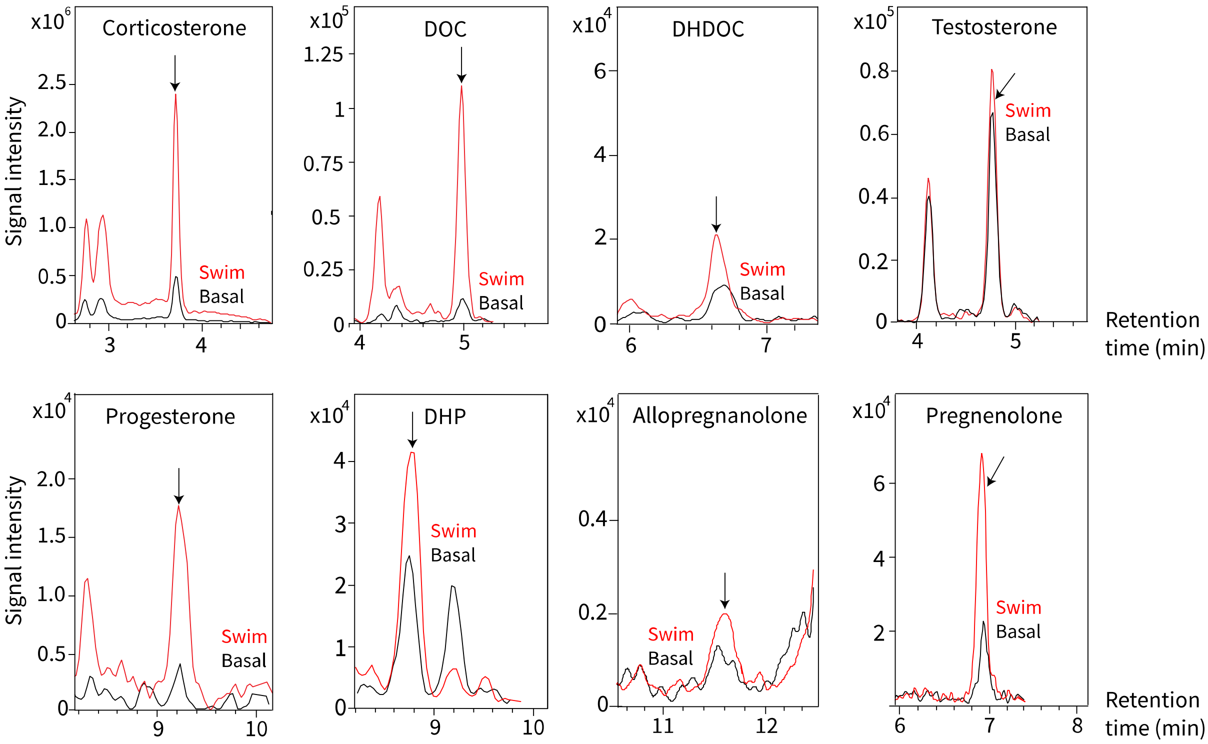

Figure S3: Representative extracted ion chromatogram of selected steroids in the male rat frontal cortex

Representative chromatograms obtained from the frontal cortex of a male swim-stressed rat (red) were overlaid on chromatograms obtained from the frontal cortex of a male non-stressed rat (black). A greater signal intensity was observed for most steroids (except testosterone) in the swim-stressed sample, indicating greater amounts of these steroids of interest in the sample.

Supplementary Figure 4

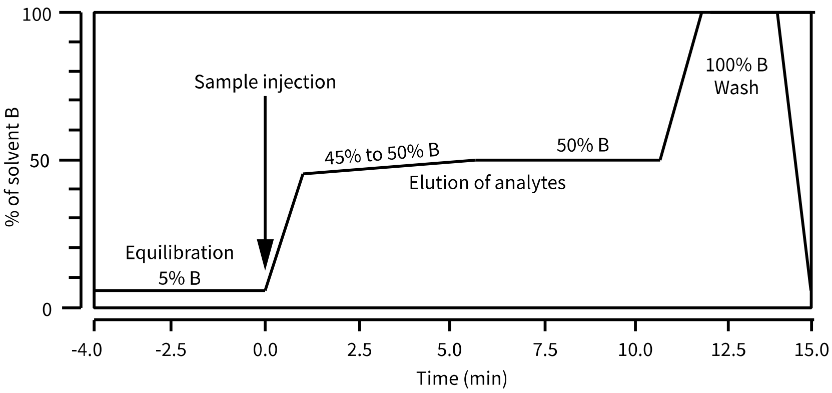

Figure S4: Gradient characteristics of high performance liquid chromatography. Solvent A: 50 mM ammonium formate pH 3.0, solvent B: methanol/0.1% formic acid. Total run time of 19 min. -4.0 min to 0 min: 5% B, 0 min to 1.0 min: 0% B to 45% B, 1.0 min to 6.0 min: 45% B to 50% B, 6.0 min to 11.0 min: 50% B, 11.0 min to 12.0 min: 50% to 100% B, 12.0 min to 14.0 min, 100% B (wash), 14.0 min – 15.0 min 100% B to 5% B.

Supplementary Table 1

Analyte	Molecular weight	Precursor ion (m/z)	Fragment ion (m/z)	Retention time (min)	Segment	Amplitude; Cut-Off
**Corticosterone**	346.5	460.2	401.1	3.5	1	117; 0.80
**Corticosterone-d4**	350.5	464.3	405.1	3.5	1	117; 0.80
**Testosterone**	288.4	402.2	343.1	4.4	1	117; 0.80
**DOC**	330.5	444.2	385.1	4.6	1	120; 0.95
**DHDOC**	332.5	446.2	387.1	6.1	2	116; 0.80
**Pregnenolone**	316.5	430.2	371.1	6.3	2	116; 0.75
**THDOC**	334.5	448.2	389.1	8.0	3	116; 0.75
**Progesterone-d9**	323.5	437.5	368.3	8.2	3	116; 0.70
**Progesterone**	314.5	428.2	369.1	8.4	3	116; 0.75
**DHP**	316.5	430.2	371.1	8.6	3	116; 0.75
**Allopregnanolone**	318.5	432.2	373.1	10.4	4	116; 0.70
**Allopregnanolone-d5**	323.5	437.6	368.3	10.5	4	120; 0.75

Table S1: **Parameters for mass spectrometric analysis of steroids and internal standards.**

Girard's T derivatisation targets carbonyl groups on steroids. Mono-derivatisation with Girard's T reagent produced a precursor ion with an addition of 114 Da to its absolute mass. Upon fragmentation by collision-induced fragmentation, a fragment ion with neutral loss of 59.1 Da (corresponding to the loss of the trimethylamine moiety) was produced, which was used for identification and quantification. Although corticosterone, DOC, DHDOC, progesterone and DHP possess two carbonyl groups, signals obtained at the expected m/z for di-derivatised compounds were negligible as compared to these mono-derivatised compounds. While isomers of allopregnanolone and testosterone were not investigated in this study, it is unlikely that these will co-elute and cause interference, as Girard's T derivatisation is expected to effectively separate these stereoisomers, as demonstrated by Dury *et al* (2016) (Girard's T separated pregnanolone, allopregnanolone, epipregnanolone and epiallopregnanolone without any interference or riding peaks) and Tamae *et al* (2013) (Girard's T separated DHEA, testosterone and epitestosterone). Moreover, the peak shapes and retention times of each individual analyte in every sample were visually inspected to ensure they conformed closely to those of the standard calibrants.

Supplementary Table 2

Analyte	QC Level	Intra-assay variability (%)	Inter-assay variability (%)	Accuracy (%)
**Corticosterone**	Low	14.0	10.6	114
(ISTD: Corticosterone-d4)	Medium	9.8	5.2	103
R^2: 0.998	High	10.0	7.0	99
**DOC**	Low	4.3	12.8	100
(ISTD: Corticosterone-d4)	Medium	3.7	5.7	95
R^2: 0.998	High	6.0	5.0	92
**DHDOC**	Low	0.9	14.0	91
(ISTD: Progesterone-d9)	Medium	17.9	23.3	76
R^2: 0.997	High	10.2	24.3	62
**THDOC**	Low	8.9	4.4	95
(ISTD: Progesterone-d9)	Medium	19.4	17.7	67
R^2: 0.994	High	12.3	16.7	63
**Progesterone**	Low	7.5	11.7	125
(ISTD: Progesterone-d9)	Medium	14.2	6.5	125
R^2: 0.998	High	7.3	22.5	108
**DHP**	Low	9.0	11.2	101
(ISTD: Progesterone-d9)	Medium	9.0	15.5	98
R^2: 0.998	High	4.2	28.0	85
**Allopregnanolone**	Low	2.7	10.7	117
(ISTD: Allopregnanolone-d5)	Medium	2.4	2.9	119
R^2: 0.996	High	5.2	5.7	110
**Pregnenolone**	Low	5.7	12.6	116
(ISTD: Allopregnanolone-d5)	Medium	3.3	13.3	132
R^2: 0.998	High	6.9	15.1	118
**Testosterone**	Low	4.5	18.3	97
(ISTD: Corticosterone-d4)	Medium	9.8	5.3	84
R^2: 0.997	High	2.1	11.7	79

Table S2: Assay performance characteristics

Assay precision was examined using low (500 pg/ml), medium (2000 pg/ml) or high (8000 pg/ml) concentrations of quality control standards in 4% BSA. Three independent samples from each concentration were used to assess intra-assay variability (% CV obtained from three independent samples in a single run, against a single calibration curve) and inter-assay variability (% CV of three independent samples across three different runs, against three different calibration curves). ISTD: internal standards, % CV: % coefficient of variation, calculated by the formula: (standard deviation of samples)/(mean of samples) x 100%. Accuracy refers to how closely the values reflect the actual concentration of steroids in the sample, and was calculated from the means of three independent samples using the formula: (known concentration)/(actual concentration) x 100%. R^2 values indicate the linearity of the calibration curves.

Supplementary Table 3

**Corticosterone**	**Main effects**			**Interactions**
	Sex	Acute Stress	Prenatal Stress	
Plasma	n.s.	$F_{1,70}$=49.3, ***	n.s.	n.s.
Frontal Cortex	$F_{1,70}$=5.43, *	$F_{1,70}$=44.8, ***	n.s.	n.s.
Hypothalamus	$F_{1,70}$=10.7, **	$F_{1,70}$=49.7, ***	n.s.	n.s.
Hippocampus	n.s.	$F_{1,70}$=87.3, ***	n.s.	Prenatal Stress x Sex, $F_{1,70}$=5.93, *
Amygdala	n.s.	$F_{1,70}$=109.8, ***	n.s.	n.s.
Brainstem	$F_{1,70}$=14.5, ***	$F_{1,70}$=37.9, ***	n.s.	n.s.

**DOC (log transformed)**	**Main effects**			**Interactions**
	Sex	Acute Stress	Prenatal Stress	
Plasma	$F_{1,70}$=10.5, **	$F_{1,70}$=74.3, ***	n.s.	Acute Stress x Sex, $F_{1,70}$=4.37, *
Frontal Cortex	$F_{1,70}$=14.8, ***	$F_{1,70}$=78.0, ***	n.s.	n.s.
Hypothalamus	$F_{1,70}$=60.5, ***	$F_{1,70}$=55.4, ***	n.s.	n.s.
Hippocampus	$F_{1,70}$=102.6, ***	$F_{1,70}$=21.4, ***	n.s.	n.s.
Amygdala	$F_{1,70}$=20.1, ***	$F_{1,70}$=100.6, ***	n.s.	n.s.
Brainstem	$F_{1,70}$=36.4, ***	$F_{1,70}$=64.6, ***	n.s.	n.s.

**DHDOC (log transformed)**	**Main effects**			**Interactions**
	Sex	Acute Stress	Prenatal Stress	
Plasma	$F_{1,69}$=219, ***	n.s.	n.s.	n.s.
Frontal Cortex	$F_{1,69}$=119.9, ***	$F_{1,69}$=39.1, ***	n.s.	n.s.
Hypothalamus	n.s.	$F_{1,69}$=29.1, ***	n.s.	n.s.
Hippocampus	$F_{1,69}$=76.3, ***	$F_{1,69}$=65.0, ***	n.s.	n.s.
Amygdala	$F_{1,69}$=61.3, ***	$F_{1,69}$=41,3. ***	n.s.	n.s.
Brainstem	$F_{1,69}$=81.6, ***	$F_{1,69}$=42.3, ***	n.s.	n.s.

**THDOC (log transformed)**	**Main effects**			**Interactions**
	Sex	Acute Stress	Prenatal Stress	
Plasma	$F_{1,69}$=52.4, ***	$F_{1,69}$=15.2, ***	n.s.	Acute Stress x Sex, $F_{1,69}$=6.67, *
Frontal Cortex	$F_{1,69}$=224.0, ***	$F_{1,69}$=26.6, ***	n.s.	n.s.
Hypothalamus	n.s.	$F_{1,69}$=29.9, ***	n.s.	n.s.
Hippocampus	$F_{1,69}$=57.2, ***	$F_{1,69}$=16.2, ***	n.s.	n.s.
Amygdala	$F_{1,69}$=32.6, ***	$F_{1,69}$=19.7, ***	n.s.	n.s.
Brainstem	$F_{1,69}$=48.2, ***	$F_{1,69}$=28.3,***	n.s.	n.s.

**Progesterone (log transformed)**	**Main effects**			**Interactions**
	Sex	Acute Stress	Prenatal Stress	
Plasma	$F_{1,70}$=161.7, ***	$F_{1,70}$=27.5, ***	n.s.	Acute Stress x Sex, $F_{1,70}$=2.84, p=0.09
Frontal Cortex	$F_{1,70}$=224.0, ***	$F_{1,70}$=43.6, ***	n.s.	Acute Stress x Sex, $F_{1,70}$=4.92, *
Hypothalamus	$F_{1,70}$=73.5, ***	$F_{1,70}$=37.9, ***	n.s.	Acute Stress x Sex, $F_{1,70}$=8.12, **
Hippocampus	$F_{1,70}$=194.9, ***	$F_{1,70}$=44.1, ***	n.s.	Acute Stress x Sex, $F_{1,70}$=15.0, ***
Amygdala	$F_{1,70}$=198.1, ***	$F_{1,70}$=39.5, ***	n.s.	Acute Stress x Sex, $F_{1,70}$=5.04, *
Brainstem	$F_{1,70}$=239.1, ***	$F_{1,70}$=52.7, ***	n.s.	Acute Stress x Sex, $F_{1,70}$=10.2, **

**DHP (log transformed)**	**Main effects**			**Interactions**
	Sex	Acute Stress	Prenatal Stress	
Plasma	$F_{1,70}$=99.3, ***	n.s.	n.s.	n.s.
Frontal Cortex	$F_{1,70}$=311.,8, ***	$F_{1,70}$=20.1, ***	n.s.	n.s.
Hypothalamus	$F_{1,70}$=53.5, ***	$F_{1,70}$=17.1, ***	n.s.	Acute Stress x Sex, $F_{1,70}$=5.54, *
Hippocampus	$F_{1,70}$=229.7, ***	$F_{1,70}$=8.53, **	n.s.	Acute Stress x Sex, $F_{1,70}$=4.74, *
Amygdala	$F_{1,70}$=178.9, ***	$F_{1,70}$=11.5, **	n.s.	Acute Stress x Sex, $F_{1,70}$=4.78, *
Brainstem	$F_{1,70}$=250.2, ***	$F_{1,70}$=33.1, ***	n.s.	Acute Stress x Sex, $F_{1,70}$=10.3, **

**Allopregnanolone (log transformed)**	**Main effects**			**Interactions**
	Sex	Acute Stress	Prenatal Stress	
Plasma	$F_{1,70}$=647.6, ***	$F_{1,70}$=4.1, ***	n.s.	Acute Stress x Sex, $F_{1,70}$=6.5, *
Frontal Cortex	$F_{1,70}$=297.,1, ***	$F_{1,70}$=20.3, ***	n.s.	n.s.
Hypothalamus	$F_{1,70}$=338.8, ***	$F_{1,70}$=28.3, ***	n.s.	Acute Stress x Sex, $F_{1,70}$=4.94, *
Hippocampus	$F_{1,70}$=335.7, ***	$F_{1,70}$=14.8, ***	n.s.	Acute Stress x Sex, $F_{1,70}$=10.4, **
Amygdala	$F_{1,70}$=230.7, ***	$F_{1,70}$=13.3, ***	n.s.	n.s.
Brainstem	$F_{1,70}$=374.1, ***	$F_{1,70}$=38.8, ***	n.s.	Acute Stress x Sex, $F_{1,70}$=12.8, ***

**Pregnenolone (log transformed)**	**Main effects**			**Interactions**
	Sex	Acute Stress	Prenatal Stress	
Plasma	$F_{1,70}$=407.8,***	$F_{1,70}$=59.0, ***	n.s.	n.s.
Frontal Cortex	$F_{1,70}$=103.9, ***	$F_{1,70}$=111.4, ***	n.s.	n.s.
Hypothalamus	$F_{1,70}$=87.3, ***	$F_{1,70}$=95.8	n.s.	n.s.
Hippocampus	$F_{1,70}$=123.8, ***	$F_{1,70}$=121.0, ***	n.s.	n.s.
Amygdala	$F_{1,70}$=123.0, ***	$F_{1,70}$=185.9, ***	n.s.	n.s.
Brainstem	$F_{1,70}$=234.0,***	$F_{1,70}$=99.3, ***	n.s.	n.s.

**Testosterone (log transformed)**	**Main effects**			**Interactions**
	Sex	Acute Stress	Prenatal Stress	
Plasma	$F_{1,71}$=499.1, ***	n.s.	n.s.	n.s.
Frontal Cortex	$F_{1,71}$=430.7, ***	n.s.	n.s.	n.s.
Hypothalamus	$F_{1,71}$=153.7, ***	n.s.	n.s.	n.s.
Hippocampus	$F_{1,71}$=447.3, **	n.s.	n.s.	n.s.
Amygdala	$F_{1,71}$=426.0, ***	n.s.	n.s.	n.s.
Brainstem	$F_{1,71}$=133.6, ***	n.s.	n.s.	n.s.

Table S3: Results of three-way ANOVA analysing the effects of acute stress x prenatal stress x sex on neuroactive steroid concentrations. Following a normality test, three-way ANOVAs were performed on natural log transformed data (except for corticosterone), with sex, acute stress and prenatal stress as the main factors. Robust main effects of sex were detected for virtually all analytes in all tissues, indicating differences in absolute concentrations of steroids. Two-way interactions (acute stress x sex) were present, in particular for progesterone, DHP and allopregnanolone. This was

mainly a result of the differential response in progesterone and its metabolites following swim stress in the female offspring. There were no main effects of prenatal stress observed for any of the analytes in any of the regions, nor were there any three-way interactions for any of the analytes examined. *P<0.05, **P<0.01, ***P<0.001. n.s.: not significant.

References

Dury AY, YY Ke, Labrie F. Precise and accurate assay of pregnenolone and five other neurosteroids in monkey brain tissue by LC-MS/MS*. Steroids* 2016;113:64-70.

Tamae D, Byrns M, Marck B, Mostaghel E, Nelson PS, Lange P, Lin D, Taplin ME, Balk S, Ellis W, True L, Vessella R, Montgomery B, Blair IA, Penning TM. Development, validation and application of a stable isotope dilution liquid chromatography electrospray ionization/selected reaction monitoring/mass spectrometry (SID-LC/ESI/SRM/MS) method for quantification of keto-androgens in human serum*. J Steroid Biochem Mol Biol.* 2013;138:281-289.
